# Tumor-associated macrophages promote PD-L1 expression in tumor cells by regulating PKM2 nuclear translocation in pancreatic ductal adenocarcinoma

**DOI:** 10.1038/s41388-021-02133-5

**Published:** 2021-12-03

**Authors:** Qing Xia, Jing Jia, Chupeng Hu, Jinying Lu, Jiajin Li, Haiyan Xu, Jianchen Fang, Dongju Feng, Liwei Wang, Yun Chen

**Affiliations:** 1 Department of Oncology, Renji Hospital, School of Medicine, Shanghai Jiao Tong University, Shanghai, 200127 China; 2 Medical Center for Digestive Diseases, The Second Affiliated Hospital of Nanjing Medical University, Nanjing, 210011 Jiangsu China; 3 Research Center for Clinical Oncology, Jiangsu Cancer Hospital, Jiangsu Institute of Cancer Research, Nanjing Medical University Affiliated Cancer Hospital, Nanjing, 210018 Jiangsu China; 4 Department of Immunology, Key Laboratory of Human Functional Genomics of Jiangsu Province, Nanjing Medical University, Nanjing, 211166 Jiangsu China; 5 Jiangsu Key Lab of Cancer Biomarkers, Prevention and Treatment, Collaborative Innovation Center for Cancer Personalized Medicine, Nanjing Medical University, Nanjing, 211166 Jiangsu China; 6 Department of Nuclear Medicine, Renji Hospital, School of Medicine, Shanghai Jiao Tong University, Shanghai, 200127 China; 7 State Key Laboratory of Oncogenes and Related Genes, Shanghai Cancer Institute, Department of Oncology, Renji Hospital, School of Medicine, Shanghai Jiao Tong University, Shanghai, 200127 China; 8 Department of Pathology, Renji Hospital, School of Medicine, Shanghai Jiao Tong University, Shanghai, 200127 China

**Keywords:** Tumour immunology, Tumour immunology

## Abstract

In many types of cancer, tumor cells prefer to use glycolysis as a major energy acquisition method. Here, we found that the ^18^fluoro-deoxyglucose (FDG) positron emission tomography (PET)/computed tomography (CT)-based markers were positively associated with the expression of programmed cell death ligand 1 (PD-L1), pyruvate kinase M2 (PKM2), both of which indicate poor prognosis in patients with pancreatic ductal adenocarcinoma (PDAC). However, the regulatory mechanism of PD-L1 remains elusive. In this study, we confirmed that transforming growth factor-beta1 (TGF-β1) secreted by tumor-associated macrophages (TAMs) was a key factor contributing to the expression of PD-L1 in PDAC cells by inducing the nuclear translocation of PKM2. Using co-immunoprecipitation and chromatin immunoprecipitation assays, we demonstrated that the interaction between PKM2 and signal transducer and activator of transcription 1 (STAT1) was enhanced by TGF-β1 stimulation, which facilitated the transactivation of *PD-L1* by the binding of PKM2 and STAT1 to its promoter. In vivo, PKM2 knockdown decreased PD-L1 expression in PDAC cells and inhibited tumor growth partly by promoting natural killer cell activation and function, and the combination of PD-1/PD-L1 blockade with PKM2 knockdown limited tumor growth. In conclusion, PKM2 significantly contributes to TAM-induced PD-L1 overexpression and immunosuppression, providing a novel target for immunotherapies for PDAC.

## Introduction

Immunotherapies based on immune checkpoint (ICP) blockades are a promising therapeutic strategy for patients with advanced liquid and solid tumors [1]. Unfortunately, progress has not extended to immunotherapies for pancreatic ductal adenocarcinoma (PDAC), with clinical trials assessing immune-based approaches failing to show efficacy in newly diagnosed and recurrent tumors [2]. A major factor involved in initial resistance to ICP inhibitors is the lack or paucity of tumor T cell infiltration, characterizing the so-called “cold tumors” [3]. The programmed cell death ligand 1 (PD-L1)/programmed cell death protein 1 (PD-1) signaling is a major co-inhibitory checkpoint pathway that controls cytotoxic adaptive immune responses, whereas the high expression of PD-L1 in PDAC is associated with poor prognosis and clinical response [4], highlighting the need for a better understanding of the processes that regulate PD-L1 expression.

Aerobic glycolysis is considered the main metabolic pathway in PDAC that exhibits metabolic plasticity [5]. Intertumoral metabolic heterogeneity affects the responses to PD-1 inhibitor therapy, and the balance between distinct metabolic pathways may influence PDAC outcomes and image metabolic heterogeneity in cancer [6]. The M2 isoform of pyruvate kinase (PKM2) catalyzes the final and rate-limiting reactions in the glycolytic pathway. PKM2 exists primarily as an enzymatically inactive monomer or dimer, a stage when PKM2 can translocate to the nucleus. Recent studies have identified important roles of PKM2 as a coactivator of hypoxia-inducible factor 1-alpha, thereby regulating the expression of PD-L1 in primary macrophages and further contributing to tumorigenesis [7].

PDAC has an immunosuppressive tumor microenvironment (TME) characterized by the presence of tumor-associated macrophages (TAMs), which are an M2-like phenotype and regulate many critical processes, including the promotion of tumor growth and metastasis, induction of angiogenesis, and immune suppression [8]. TAMs or stromal cell-derived factors, such as transforming growth factor-beta1 (TGF-β1), are well-established modifiers of the pancreatic TME that attenuates tumor response to PD-L1 blockade [9]. Clinically, during the early phase of PDAC development, TGF-β1 expression is inversely associated with granzyme B (GzmB), a cytolytic granule that plays a pivotal role in natural killer (NK) cell-mediated cytotoxicity against early-stage cancers [10]. To date, although much work has focused on the regulatory network of genomic aberrations, inflammatory signaling, and posttranslational modulation that determines PD-L1 levels [11, 12], the mechanism by which TAM-mediated PKM2 translocation in tumors induces immunosuppression in PDAC is unclear.

Here, we found the first direct link between two of the central players in cancer, PKM2 and PD-L1, providing new mechanistic insight into the regulation of PD-L1 expression by PKM2 in tumor cells that further influences NK cell activation, but more dependently on TAM-derived TGF-β1. Our findings may provide novel therapeutic direction in the growing field of combination immunotherapy for cancer.

## Materials and methods

### Clinical samples

Clinical tissue samples from patients with PDAC who had undergone surgical resections were collected between February 2017 and December 2018 at Renji Hospital affiliated to Shanghai Jiaotong University School of Medicine. None of the patients had received antitumor therapy before surgery. The patients’ clinical characteristics were classified according to the guidelines of the Union for International Cancer Control (TNM staging). All experiments were performed in compliance with government policies and the Helsinki Declaration. The individuals were informed about the study and gave consent before specimen collection. The study was approved by the Ethics Committee of the Affiliated Renji Hospital of Shanghai Jiao Tong University. The clinical and pathological features of the involved patients in this study are presented in Supplementary Table 1. All available histological slides were reviewed by a pathologist specializing in pancreatic diseases, and the following items were systematically assessed: Immunohistochemistry (IHC); immunofluorescence staining; PET/CT image analysis; and TNM stage.

For further details regarding the materials and methods, please refer to the Supplementary information.

## Results

### Clinical correlation between PD-L1 and PKM2 expression to predict poor prognosis in patients with PDAC

PD-L1 protein and mRNA levels are elevated in PDAC tissues [2]. To determine the clinical utility of strategies targeting the PD-L1 pathway, the correlation analysis for PD-L1 and clinical outcomes (*n* = 176) in The Cancer Genome Atlas (TCGA) cohort were explored, indicating high PD-L1 expressions mark the poor survival of patients with PDAC (Fig. S1a). Moreover, we found that the high expression levels of PD-L1 could discriminate prognostic groups in both univariate and multivariate analyses (Fig. S1b, c).

**Fig. 1:**
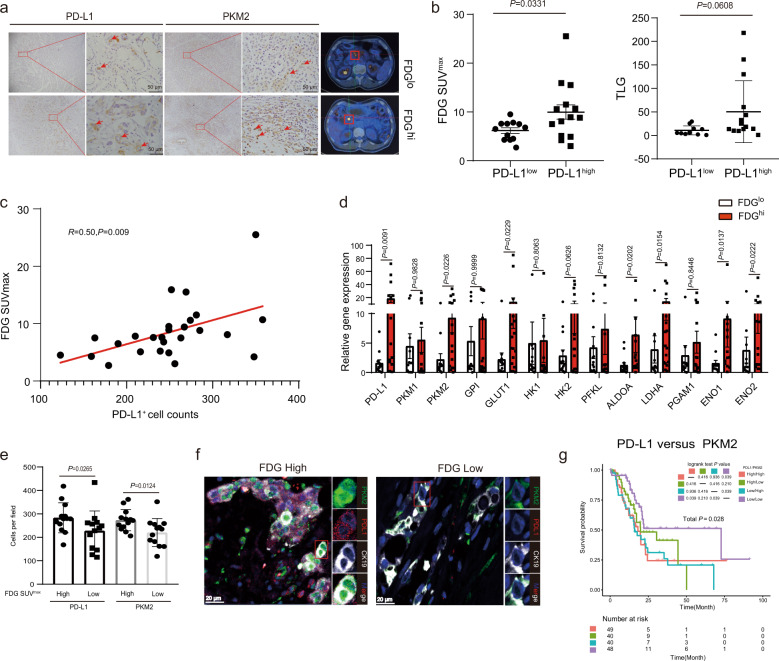
PD-L1 is correlated with PKM2, which predicts a worse prognosis in patients with PDAC. **a** Representative PET/CT images for FDG uptake (right) and immunostaining of PD-L1 and PKM2 (left) from paired tumors of patients with PDAC. Right, red box indicated tumor locations. Left, red arrowheads showed PD-L1- or PKM2-positive tumor cells. Scale bar, 50 μm. **b** Glycolysis for PD-L1^lo^ or PD-L1^hi^ PDAC tumors (*n* = 26). Glycolysis was analyzed as FDG maximal standardized uptake value (FDGSUV_max,_ left) and total lesion glycolysis (TLG, right) values. The median value of the counts of the PD-L1-positive tumor cells were used as a cutoff to divide PD-L1^lo^ vs. PD-L1^hi^ tumors. **c** The correlation between FDG SUV_max_ values and PD-L1-positive tumor cells in 26 patients with PDAC. **d** The mRNA levels of the genes of glucose metabolism in PDAC tissues with FDG^hi^ or FDG^lo^ were analyzed using RT-PCR. The median value of the FDG uptake values was used as a cutoff to divide FDG^hi^ vs. FDG^lo^ tumors. **e** Correlations of PD-L1 and PKM2-positive tumor cells densities with FDG SUV_max_ values expression in 26 patients with PDAC. Counts of PD-L1- and PKM2-positive tumor cells in an area of tumor per 0.95 mm^2^ were randomly chosen and calculated. **f** Representative immunofluorescence staining of PKM2+ cells (green), PD-L1+ cells (red), and CK19+ cells (white) in PDAC tissues with FDG^hi^ or FDG^lo^. Scale bar, 20 μm. **g** The overall survival of patients with PDAC based on PKM2 and PD-L1 expression according to the TCGA dataset. Analysis was performed using Kaplan–Meier estimates and two-sided log-rank tests. *P*-values were obtained using the Mann–Whitney *U*-test in (**b**, **d**, **e**). *P-*values are indicated in each plot.

Tumor glycolysis under hypoxia is considered an essential factor for immunosuppressive effects by regulating PD-L1 in tumor cells and immunocytes [13]. To explore whether tumor glycolysis facilitates PD-L1 expression, we examined PD-L1 by immunostaining 26 pairs of biopsy samples obtained from the patients with PDAC, who had also been imaged using fluorine-18 fluorodeoxyglucose (^18^F-FDG) positron emission tomography (PET) as an indicator for metabolic activity of viable tumor cells. We observed a relationship between FDG uptake and PD-L1 expression in tumors (Figs. 1a and S1d), as measured by both FDG maximal standardized uptake value (SUVmax) and total lesion glycolysis (TLG), which comprehensively reflect both metabolic activity and tumor volume (Fig. 1b, c). To identify the key glycolytic genes that might be important for the aberrant regulation of PD-L1 expression, high-FDG and low-FDG PDAC samples were compared. The results revealed significant differences in several metabolic enzymes, including PKM2 and lactate dehydrogenase A (LDHA), by performing real-time quantitative reverse transcription polymerase chain reaction (qRT-PCR) (Fig. 1d). The PKM2 mRNA levels of PDAC tissues were significantly higher than those in non-tumor pancreas tissues from TCGA and GTEx database (*P* < 0.05) (Fig. S1e). Kaplan-Meier survival analysis showed a higher rate of survival among patients with lower PKM2 expression as compared with those with higher PKM2 expression (*P* = 0.0054; hazard ratio (HR) = 1.633) (Fig. S1f). Furthermore, we observed that patients with PDAC with high-FDG levels exhibited high PD-L1 expression (*P* = 0.0265) and high PKM2 expression (*P* = 0.0124) (Fig. 1e). IIHC results revealed that PKM2 was mainly detected in the nuclei of tumor cells in high-FDG tissues (Fig. 1a). Furthermore, we concluded that using the TCGA data of the patients with PDAC analyzed, PD-L1 expression was positively related to the genes of glucose metabolism, including hexokinase 1, phosphoglycerate mutase 1, enolase 1, PKM2, and LDHA (Fig. S1g). Multicolor immunofluorescence staining of tumor tissue sections revealed that the expressions of PKM2 and PD-L1 were significant higher in FDG^hi^ patients than FDG^lo^ patients. Notably, the nuclear localization of PKM2 was observed in PDAC with high FDG, but hardly detected in those with low FDG (Fig. 1f). The Kaplan–Meier curves indicated that patients with high PD-L1 and PKM2 expressions in cancer cells conferred significantly worse prognosis than those with low PD-L1 and PKM2 expressions (PD-L1^hi^ + PKM2^hi^ vs. PD-L1^lo^ + PKM2^lo^; *P* = 0.039; HR, 3.019; 95% CI, 1.348–1.775) (Fig. 1g). In conclusion, PD-L1 is associated with high glycolysis activity and its combination with PKM2 predicts a worse prognosis in PDAC.

### PKM2 knockdown decreases PD-L1 expression and induces antitumor immunity in vivo

**Fig. 2:**
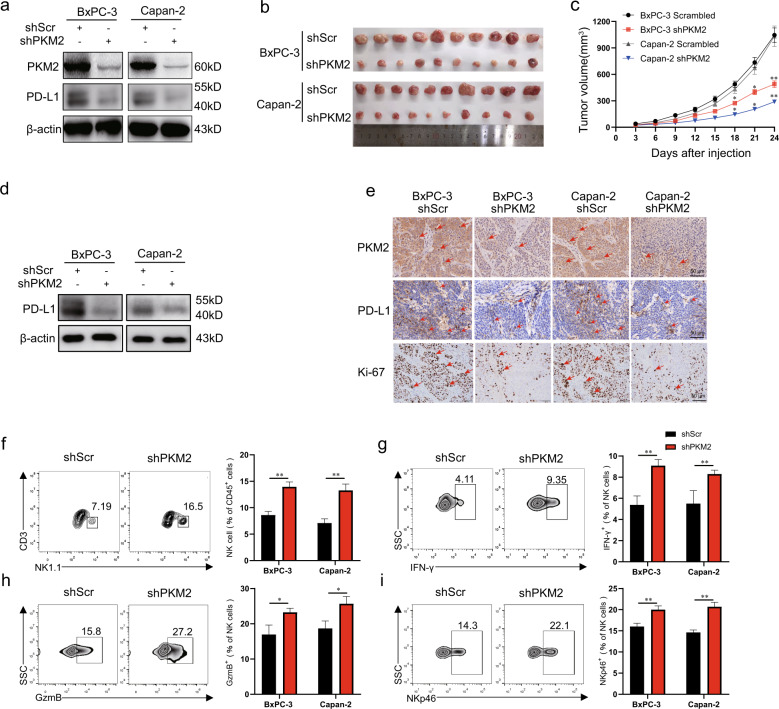
PKM2 knockdown decreases the expression of PD-L1 and inhibits tumor growth of PDAC partly by activating NK cells. **a** PKM2 expression in BxPC-3/Capan-2 cells was modified by shRNA interference, and the expression of PKM2 and PD-L1 was measured by western blotting. **b** Tumor xenografts formed by implanted BxPC-3/Capan-2 cells with different expression levels of PKM2. **c** Scramble or sh-PKM2 was stably infected into BxPC-3/Capan-2cells, which were injected into nude mice. Tumor volumes were calculated after injection every 3 days. Tumor volumes are represented as the means of tumor volumes ±SEM (*n* = 10). **d** Tumor xenografts were isolated from the nude mice on day 24, the expression level of PD-L1 in tumor tissue was detected using western blotting. **e** Representative immunohistochemical staining of PKM2, PD-L1, and Ki-67 in tumor serial sections, which were derived from xenografts in the groups of BxPC-3/Capan-2 cells with different expression levels of PKM2. Scale bar, 50 μm. Red arrowheads showed PD-L1 or PKM2-positive tumor cells. **f** Single-cell suspensions from BxPC-3/Capan-2 tumors with different expression levels of PKM2 isolated from the nude mice on day 24 were stained for flow cytometry. Representative flow cytometry plots showed NK cells from BxPC-3 tumors with different expression levels of PKM2 (left), and the percentages of tumor-infiltrating leukocyte subsets (right) in gated CD45^+^ cells were analyzed from BxPC-3/Capan-2 tumors with different expression levels of PKM2 (*n* = 10 per group). **g**–**i** Single-cell suspensions from BxPC-3/Capan-2 tumors with different expression levels of PKM2 isolated from the nude mice on day 24 were stained for flow cytometry. Representative flow cytometry plots (left) and percentages of IFN-γ^+^, GzmB^+^, and NKp46^+^ NK cell (right) in gated CD3^−^ NK1.1^+^ cells (*n* = 10). Data are shown as mean ± SEM from five mice per group and are representative of two experiments. Statistical significance was determined by the Mann–Whitney *U*-test. NS = no significance; ^**^P < 0.01.

Next, we investigated whether PKM2 regulates PD-L1 expression in human PDAC cell lines. BxPC-3 and Capan-2 cells were infected with PKM2-specific or non-targeting shRNAs. The reduction of PKM2 closely correlated with a marked reduction in PD-L1 expression of PDAC cells, whereas the scrambled (non-targeting) shRNA showed no such effect (Fig. 2a). In addition, PKM2 knockdown decreased the levels of both glucose uptake and lactate production in PDAC cells (Fig. S2a). To determine the effect of the regulation of PKM2 on PD-L1 expression and its role in the tumorigenesis of PDAC in vivo, xenograft mice models were established by subcutaneously injecting scrambled-shRNA- or PKM2-shRNA-infected BxPC-3 and Capan-2 cells. Compared with the control group, the xenograft volume decreased in the shRNA-PKM2 groups (Fig. 2b, c). The downregulated PD-L1 expression from the PDAC tissues in the shRNA-PKM2 groups was confirmed by western blotting and IHC (Fig. 2d, e). Moreover, the results of the IHC analysis revealed that the expression of proliferation marker Ki-67 was dramatically reduced in the PKM2-shRNA group in comparison with the scramble-shRNA group (Fig. 2e). Additionally, decreased proliferation of PDAC cells after infection with PKM2-shRNA was also observed by the 5-ethynyl-29-deoxyuridine (EdU) assay (Fig. S2b). There was a trend of increase of cell apoptosis after infection, but it was not significantly different from that before infection (*P* > 0.05) (Fig. S2c). Given that a substantially obvious association existed between PD-L1 expression and PKM2 levels both in vitro and in vivo, we next sought to determine whether PKM2 may induce changes in tumor-infiltrating immune cells in nude mice with PDAC by flow cytometry. We found that PDAC tumors with PKM2 knockdown had significantly more NK cells (Figs. 2f and S2d) and higher expressions of effector molecules, such as interferon (IFN)-γ and GzmB, along with NKp46 in NK cells (Figs. 2g–i and S2e–g) than control tumors; however, no changes in the cell percentage of DC cells, macrophages, and myeloid-derived suppressor cell infiltration (Fig. S2h–j) were observed in PDAC tumors with PKM2 knockdown compared with control tumors. Collectively, these results together suggested that PKM2 knockdown decreases the expression of PD-L1 and elicits potent antitumor immunity by activating NK cells.

### The nuclear translocation of PKM2 is required for TAM-induced PD-L1 expression in PDAC

**Fig. 3:**
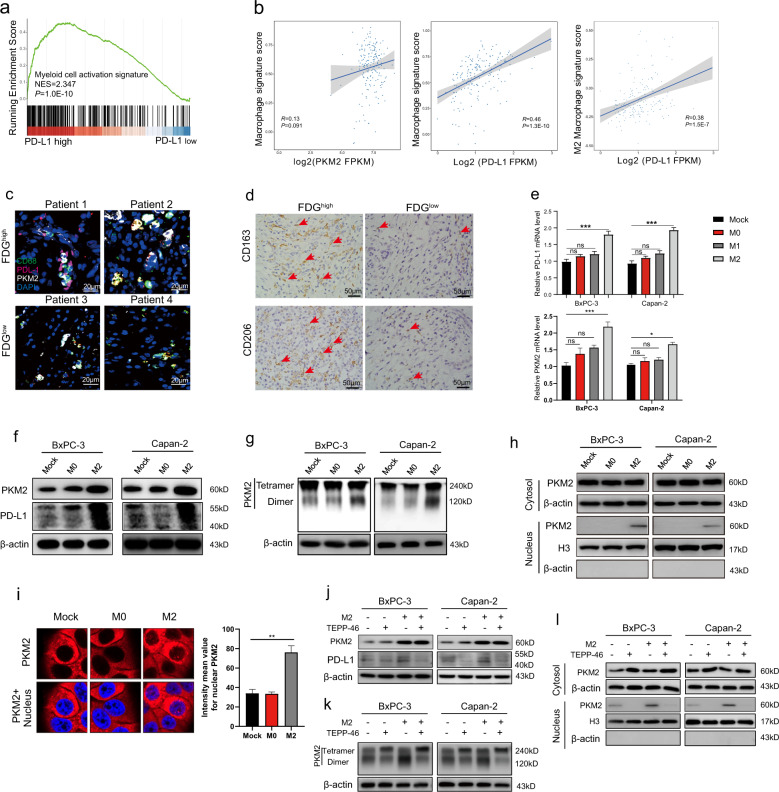
M2 macrophages upregulate PD-L1 expression in tumor cells by inducing nuclear translocation of PKM2 in PDAC. **a** GSEA of myeloid cell activation signature in PD-L1^hi^ PDAC samples vs. PD-L1^lo^ counterparts from the TCGA dataset. **b** TCGA analysis between the scoring of macrophage signature and PKM2 (left) or PD-L1 (middle). Also, correlation between M2 macrophage signature and PD-L1 was calculated in PDAC (right). **c** Representative immunofluorescence staining of CD68^+^ macrophages (green), PD-L1+ cells (red), and PKM2+ cells (white) in PDAC tissues with FDG^hi^ or FDG^lo^. Scale bar, 20 μm. **d** Representative immunostaining of CD163^+^/CD206^+^ macrophages from paired tumors of patients with PDAC with FDG^hi^ or FDG^lo^. Red arrowheads showed CD163^+^ or CD206^+^ macrophages. Scale bar, 50 μm. **e** BxPC-3/Capan-2 cells were co-cultured with M0, M1, or M2 polarized macrophages (tumor cell:macrophage = 1:2) for 48 h. Then, BxPC-3/Capan-2 cells were lysed and assayed for the expression of *PD-L1* and *PKM2* mRNA by qRT-PCR. **f** PKM2 and PD-L1 protein expression levels from whole-cell lysate of BxPC-3/Capan-2 cells co-cultured with M0/M2 macrophages were determined by western blotting. **g** BxPC-3/Capan-2 cells co-cultured with M0/M2 macrophages were cross-linked by glutaraldehyde first and then subjected to western blotting. **h** Nuclear and cytosolic lysates were prepared from BxPC-3/Capan-2 cells co-cultured with M0/M2 macrophages, followed by western blotting. **i** Subcellular localization of PKM2 in BxPC-3/Capan-2 cells co-cultured with M0/M2 macrophages. Cells were immunostained with anti-PKM2 (PKM2, red). The nucleus was marked with 4′,6-diamidino-2-phenylindole dihydrochloride (blue). **j** BxPC-3/Capan-2 cells were pretreated with TEPP-46 (10 μM) for 12 h or/and then co-cultured with M2 polarized macrophages (tumor cell:macrophage = 1:2) for 48 h. Then, PKM2 and PD-L1 protein expression levels from whole-cell lysate of BxPC-3/Capan-2 cells were determined by western blotting. **k** BxPC-3/Capan-2 cells pretreated with TEPP-46 or/and co-cultured with M2 macrophages were cross-linked by glutaraldehyde first and then subjected to western blotting. **l** Nuclear and cytosolic lysates were prepared from BxPC-3/Capan-2 cells pretreated with TEPP-46 or/and co-cultured with M2 macrophages, followed by western blotting. Data were shown as mean ± SEM from three experiments. Statistical significance was determined by a *t*-test; ^*^P < 0.05,^**^P < 0.01,^***^P < 0.001. NS = no significance.

To further investigate the immune signature of tumors with high PD-L1 expression, we identified 270 genes correlated with PD-L1 expression in PDAC tissues from TCGA (*R* ≥ 0.5; *P* ≤ 0.0001) and annotated these genes using gene ontology (GO) (Fig. S3a). Among the top 10 enrichment GO terms, eight pathways related to immune response or myeloid leukocyte activation signature were enriched intensively. Using Gene Set Enrichment Analysis (GSEA), we confirmed that PDAC tumors with high PD-L1 expression are dominantly enriched with genes indicating the myeloid cell activation signature (Fig. 3a). TAMs play a critical role in tumor glycolysis and regulating PD-L1 expression, which exhibits M2 macrophage characteristics and paves the way to carcinogenesis [14]. Pearson correlation analysis showed that the expression of both PKM2 and PD-L1 positively correlated with macrophage infiltration in the TCGA dataset. Notably, PD-L1 and PKM2 expression was highly associated with M2 macrophage signature (PD-L1, *R* = 0.38, *P* < 0.0001; PKM2, *R* = 0.26, *P* < 0.0001) (Figs. 3b and S3b). Immunofluorescence staining revealed colocalization of PKM2 and PD-L1 in the tumor tissue with high glycolysis, along with accumulation of CD68^+^ macrophages. (Figs. 3c and S3c). Moreover, IHC staining confirmed that these macrophages displayed M2 phenotype (CD163^+^/CD206^+^) within FDG^hi^ PDAC tumors (Fig. 3d and S3d).

Thus, we asked whether PKM2-induced PD-L1 dysregulation in cancer cells is involved in macrophage-mediated tumor growth. Considering that TAMs can suppress the immune response by secreting immunosuppressive factors, we co-cultured BxPC-3 or Capan-2 cells with polarized M2 macrophages in transwell experiments. M2 macrophages markedly increased the mRNA and protein expression levels of PD-L1 in PDAC cells compared with untreated or M0 macrophages (Fig. 3e, f). Meanwhile, M1 macrophages have shown similar effects on PDAC-expressing PD-L1 with M0 macrophages (Fig. 3e). Consistent with PD-L1 expression, we observed a more pronounced elevation of PKM2 mRNA and protein levels in PDAC cells after they were co-cultured with M2 macrophages (Figs. 3e, f and S3e).It has been reported that tumor cells favor PKM2 because it can switch between a highly active tetramer form and a less active dimer form. The tetramer form of PKM2 can efficiently promote glycolysis and energy production, whereas PKM2 in the dimer state can enter the nucleus to regulate gene expression [15]. Therefore, we performed a chemical cross-linking assay to determine the effects of TAMs on PKM2-induced PD-L1 upregulation. We found M2 macrophage shifts the PKM2 dimer/tetramer equilibrium to the dimeric form (Fig. 3g), which was further validated by the finding of the increased dimer/tetramer ratio of PKM2 dissociation (Fig. S3f). Western blotting of cytoplasmic and nuclear fractions showed that M2 macrophages promote PKM2 nuclear translocation (Figs. 3h and S3g). Furthermore, M2 macrophages changed the subcellular distribution of PKM2 with a marked increase in nuclear staining detected by immunofluorescence (Fig. 3i). Also, PKM2 knockdown obviously decreased fluorescence intensity in both cytoplasm and nucleus (Fig. S3h). However, inhibiting PKM2 nuclear translocation with TEPP-46, a selective PKM2 activator, attenuated PD-L1 expression (Figs. 3j and S3i). Treatment with TEPP-46, which triggered PKM2 tetramer formation, abrogated M2 macrophage-induced PKM2 enrichment in the nucleus (Figs. 3k, l and S3j–n). These results demonstrated that TAMs may modulate PD-L1 expression by increasing PKM2 nuclear localization.

### TAM-derived factor TGF-β1 induces PD-L1 expression via PKM2 nuclear translocation in PDAC cells

**Fig. 4:**
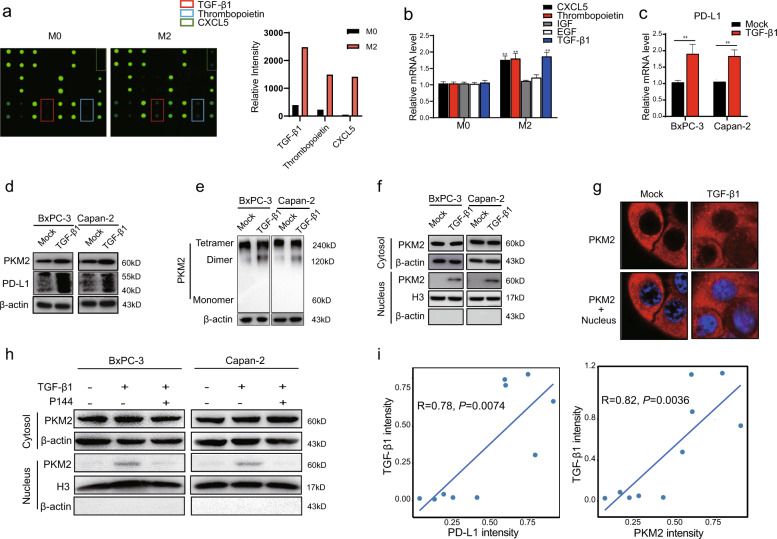
TGF-β1 secreted by M2 macrophages promotes the nuclear translocation of PKM2 in PDAC cells. **a** Cytokine antibody arrays were performed in the culture media of M0/M2 macrophages co-cultured with BxPC-3 cells (left). The relative signal intensity of indicated cytokines in M2 macrophages co-cultured with BxPC-3 cells compared with M0 macrophages co-cultured BxPC-3 cells (right). **b** Quantitative RT-PCR analysis of mRNA levels of CXCL5, Thrombopoietin, IGF, EGF, and TGF-β1 in M0/M2 macrophages co-cultured with BxPC-3/Capan-2 cells for 48 h. **c**–**g** BxPC-3/Capan-2 cells were treated with or without TGF-β1 (20 ng/mL). **c** Quantitative RT-PCR analysis of mRNA expression levels of PD-L1 in BxPC-3/Capan-2 cells. **d** PKM2 and PD-L1 protein expression levels in BxPC-3/Capan-2 cells were determined using western blotting. **e** BxPC-3/Capan-2 cells were cross-linked by glutaraldehyde first and then subjected to western blotting. **f** Nuclear and cytosolic lysates were prepared from BxPC-3/Capan-2 cells, followed by western blotting. β-actin and histone H3 were used as loading controls. **g** The subcellular localization of PKM2 in BxPC-3/Capan-2 cells. Cells were immunostained with anti-PKM2 (PKM2, red). The nucleus was marked with 4′,6-diamidino-2-phenylindole dihydrochloride (blue). The stained cells were imaged using the ZEN 3.0 (Carl Zeiss) software. **h** BxPC-3/Capan-2 cells were treated with or without TGF-β1 (20 ng/mL), and P144 (10 μM) was used to block TGF-β1. Nuclear and cytosolic lysates were prepared from BxPC-3/Capan-2 cells, followed by western blotting. **i** Correlation analyses between the expression of TGF-β1 and PKM2/PD-L1 in PDAC tissues according to western blotting (*n* = 10). Data were shown as mean ± SEM from three experiments. Statistical significance was determined by a *t*-test; ^*^P < 0.05,^**^P < 0.01,^***^P < 0.001.

To identify the TAM-secreted factor responsible for PD-L1 upregulation in tumors, we harvested supernatants from the co-culture systems and exposed them to a cytokine (human) antibody array. We observed that TGF-β1 was remarkably released, although several other cytokines were observed, including Thrombopoietin and CXCL5, whose expressions were upregulated in M2 macrophages co-cultured with BxPC-3 cells compared with M0 macrophages (Fig. 4a). Notably, the levels of TGF-β1 in M2 macrophages co-cultured with BxPC-3 cells were significantly higher than those with normal pancreatic HPNE cells (Fig. S4b). Also, there was no alteration in TGF-β1 production at low level when HPNE cells were co-cultured with M0 or M2 macrophages (Fig. S4a, b). However, there was no significant difference in the TGF-β1 level between the co-culture supernatant of BxPC-3 and HPNE cells co-cultured with TGF-β1-knockdown M2 macrophages, indicating that TGF-β1 was the key cytokine produced by M2 macrophage (Fig. S4b, c). Consistent with antibody array data, qRT-PCR confirmed that co-culture of macrophages with PDAC cells increased the aforementioned cytokines associated with M2 phenotype (Fig. 4b). Furthermore, PD-L1 expression was not upregulated in the PDAC cells co-cultured with TGF-β1-knockdown M2 macrophages, indicating that the effect of M2 macrophages on the PD-L1 expression of PDAC cells was mediated by TGF-β1 (Fig. S4d). Therefore, we treated PDAC cells with TGF-β1 and found that the mRNA and protein expression levels of PD-L1 were significantly enhanced (Figs. 4c, d and S4e). To determine whether PKM2 contributes to PD-L1 upregulation via the TGF-β1 signaling pathway, total cell lysates were subjected to chemical cross-linking assay after TGF-β1 treatment. Western blotting showed that TGF-β1 increases total PKM2 expression and the dimer/tetramer ratio in PDAC cells (Figs. 4d, e and S4f, g). These results were further supported by the detection of nuclear PKM2 enrichment in the subcellular fraction analysis and immunofluorescence (Figs. 4f, g and S4h). However, the increase of the nuclear PKM2 expression in PDAC cells induced by M2 macrophages was eliminated when the cells were treated with TGF-β1 inhibitor P144 (Fig. 4h).

It has been reported that TGF-β1 signaling could be activated through noncanonical pathways in the Smad4-null pancreatic cancer cell line BxPC-3 [16–18]. A positive correlation between PD-L1 and phosphorylated Smad2 expression was found in non-small-cell lung cancer (NSCLC), in which TGF-β1 was found to upregulate PD-L1 gene transcription in a Smad2-dependent manner rather than Smad4 [19]. Interestingly, we observed that TGF-β1 significantly increased the expression of PD-L1 through phosphorylated Smad2 (p-Smad2), but not p-Smad3 (Fig. S4i). However, silencing of Smad2 could effectively abrogate TGF-β1-induced upregulation of PD-L1, suggesting that TGF-β1/Smad2 signal pathway plays a crucial role in PKM2-mediated PD-L1 expression in BxPC-3 cells (Fig. S4j). In addition, TGF-β2 and TGF-β3 have no effect on the expression levels of both PKM2 and PD-L1 in PDAC cells (Fig. S4k). Importantly, we noted that high PD-L1 and PKM2 expression were associated with high level of TGF-β1 in PDAC tumor tissues (Figs. 4i and S4l). Likewise, in TCGA database, TGF-β1 expression in PDAC patients positively correlated with expression of PD-L1 and PKM2 (TGF-β1 vs. PKM2: *R* = 0.48, *P* = 1.2 × 10^−11^; TGF-β1 vs. PD-L1: *R* = 0.24, *P* = 0.0015) (Fig. S4m). These results indicated that TGF-β1 was a major inflammatory factor secreted by M2 macrophages that upregulated PD-L1 expression by the nuclear translocation of PKM2.

### Nuclear translocation of PKM2 and phosphorylation of STAT1 are involved in TGF-β1-induced PD-L1 upregulation

**Fig. 5:**
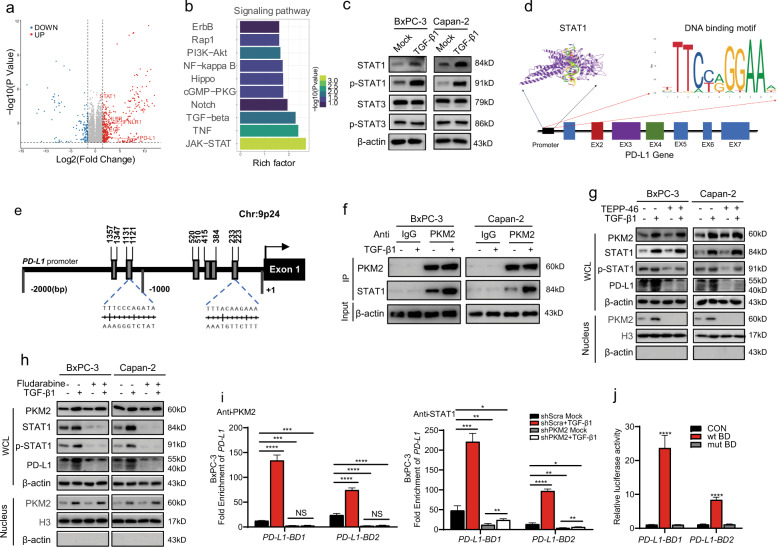
TGF-β1 induces PD-L1 expression by enhancing the nuclear translocation of PKM2 and phosphorylation of STAT1. **a** The mRNA expression profiles of BxPC-3 cells treated with (*n* = 3) or without (*n* = 3) TGF-β1 (20 ng/mL) were compared using RNA-seq analysis. Volcano plot of upregulated or downregulated genes in BxPC-3 cells treated with or without TGF-β1. Significance was determined as *P*-value < 0.05 and fold change > 2. **b** The top 10 upregulated signal transduction pathways in KEGG enrichment analysis in TGF-β1-treated BxPC-3 cells ranked by rich factor. **c** BxPC-3/Capan-2 cells were treated with or without TGF-β1 (20 ng/mL) for 12 h. STAT1, p-STAT1, STAT3, and p-STAT3 protein expression levels in BxPC-3/Capan-2 cells were determined using western blotting. **d** The representative STAT1 DNA-binding motif was predicted using the following website: https://jaspar.genereg.net. **e** The schematic structure of the human *PD-L1* promoter with potential STAT1-binding sites. **f** Immunoprecipitation analysis of indicated protein complexes in BxPC-3/Capan-2 cells following treatment with TGF-β1 (20 ng/mL) for 12 h. **g** BxPC-3/Capan-2 cells with different expression levels of PKM2 were treated with TGF-β1 (20 ng/mL) for 12 h, and then the cells were harvested, and nuclear and cytosolic lysates were prepared, followed by western blotting. **h** BxPC-3/Capan-2 cells were pretreated with fludarabine (50 μmol/mL) for 6 h, and then the cells were washed and treated with TGF-β1 (20 ng/mL) for another 6 h. Nuclear and cytosolic lysates were prepared, followed by western blotting. β-actin and histone H3 were used as loading controls. **i** The binding of STAT1 or PKM2 to the two binding domains within a *PD-L1* promoter was analyzed using Ch-IP and qPCR in BxPC-3 cells following treatment with TGF-β1 (20 ng/mL) for 12 h. **j** Relative luciferase activities in 293T cells transfected with WT STAT1 binding domain or mutant STAT1 binding domain reporter vectors. Data are expressed as fold change relative to the luciferase activity observed in non-transfected cells. Data were shown as mean ± SEM from three experiments; statistical significance was determined by a *t*-test; ^*^P < 0.05,^**^P < 0.01,^***^P < 0.001.

Dysfunctions in TGF-β signaling significantly contribute to tumorigenesis and tumor progression [20, 21]. Then, we sought to investigate the mechanisms involved in the TGF-β1-dependent expression of PD-L1 in PDAC cells. The transcriptome profile was evaluated in the control and TGF-β1-treated BxPC-3 cells by RNA sequencing analysis. We identified the over 958 genes affected by TGF-β1, including 698 upregulated genes and 260 downregulated genes (*P* < 0.05; fold change > 2.0) (Fig. 5a). Based on the Kyoto Encyclopedia of Genes and Genomes (KEGG) functional annotation, the differentially expressed genes were significantly enriched in the JAK-STAT and TGF-β1 pathways (*P* = 0.013, rich factor = 2.71 and *P* = 0.0206, rich factor = 2.29, respectively) (Fig. 5b). In this context, the expression of several key STAT-target genes, such as IFNLR1, IFNB, IL6R, EGF, and PD-L1, was increased in response to TGF-β1 stimulation (Fig. 5a). As cytokine-induced Ser/Thr phosphorylation facilitates STAT family nuclear translocation and transactivation of downstream gene expression [22], we next examined TGF-β1-triggered phosphorylation of STAT1/3 in which their transcriptional levels were elevated, especially STAT1. We observed that TGF-β1 significantly increased phosphotyrosine-STAT1 (p-STAT1) and total STAT1, whereas no significant effect on STAT3 activation and expression (Figs. 5c and S5a, b).

The results prompted us to investigate the relationship between PKM2 and STAT1 in TGF-β1-induced upregulation of PD-L1. Given that nuclear PKM2 acts as a protein kinase which phosphorylates various protein substrates [23], we hypothesized that TGF-β1 triggers PKM2 nuclear translocation which induces STAT1 phosphorylation to regulate PD-L1 expression. To this end, the transcription factor STAT1 potentially binding to the promoter region of PD-L1 was predicted by the JASPAR database, revealing striking enrichment of motifs within protein-binding sites (Fig. 5d, e). Furthermore, an interaction between PKM2 and STAT1, which was significantly enhanced by TGF-β1, was detected in PDAC cells by co-immunoprecipitation (Co-IP) (Fig. 5f). After the inhibition of PKM2 nuclear translocation by TEPP-46, the whole process was abrogated (Figs. 5g and S5c–g). In addition, fludarabine, a STAT1 activation inhibitor, significantly decreased PD-L1 protein levels but did not alter the expression of PKM2, indicating that PKM2 acted on the downstream effector STAT1 signaling (Figs. 5h and S5h–l). Next, chromatin immunoprecipitation (Ch-IP) assays were used to determine whether the coactivator of PKM2 and p-STAT1 promote the transcription of PD-L1. DNA bound to PKM2 and p-STAT1 was pulled down in a Ch-IP assay. The DNA was PCR-amplified with primers specific for the two binding domains of PD-L1 promoter and confirmed that PKM2 and STAT1 indeed demonstrated strong binding to PD-L1 upon TGF-β1 treatment but were severely impaired by PKM2 knockdown (Figs. 5i and S5m). For functional analysis of the binding sites, we constructed luciferase reporter plasmids containing the WT *PD-L1* promoter STAT1-binding motifs and mutant ones. Luciferase expression activity demonstrated that the inserted WT STAT1-binding domains were active in 293T cells (Fig. 5j), with STAT1 upregulating the expression of *PD-L1* by directly binding to the promoter-specific motifs from human PDAC cells.

### PKM2-mediated enhancement of PD-L1 expression in PDAC is inversely related to antitumor immunity of NK cells

**Fig. 6:**
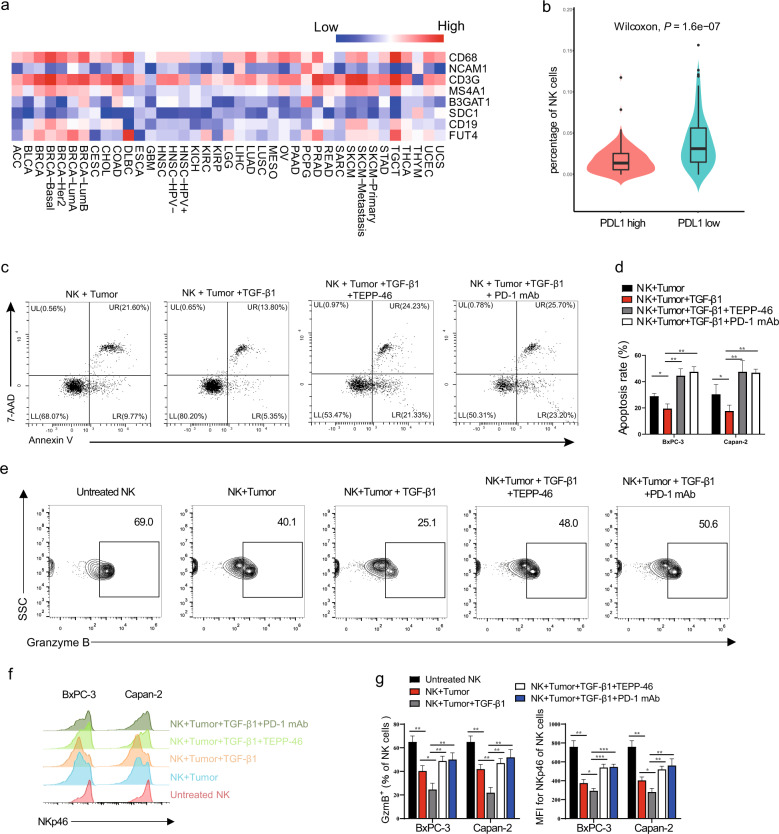
TGF-β1-regulated PD-L1 expression mediated by PKM2 in PDAC cells affects the activation and killing function of NK cells in vitro. **a** Correlations between PD-L1 and indicated genes were analyzed in patients with 32 types of cancer samples from the TCGA dataset. **b** Correlations of NK cell percentage in tumors with PD-L1 expression of patients with PDAC. **c**–**g** NK cells were isolated from PBMCs of healthy volunteers using a human NK cell isolation kit (Miltenyi Biotec). BxPC-3/Capan-2 cells were pretreated with TGF-β1 (20 ng/mL), with or without TEPP-46 (10 μM), for 12 h and washed, and then co-cultured with isolated NK cells (tumor cell:NK cell = 1:5) for another 12 h. In some instances, anti-PD-1 monoclonal antibody (20 μg/mL) was added into the co-culture system. **c**, **d** Conditioned tumor cells were harvested for apoptosis analysis by flow cytometry (**c**). The apoptosis rate of BxPC-3/Capan-2 cells was shown in (**d**). **e**–**g** Conditioned NK cells were harvested and stained for flow cytometry. A representative example of (**e**) granzyme B and (**f**) NKp46 staining on conditioned NK cells by flow cytometry. The percentage of granzyme B and NKp46 in NK cells were shown in (**g**). Data were shown as mean ± SEM from three experiments; statistical significance was determined by a *t*-test; ^*^P < 0.05,^**^P < 0.01,^***^P < 0.001.

Studies have shown that PD-L1-transduced tumor cells suppress immune resistance and that the removal of NK cells abrogates antitumor efficacy of PD-1 blockade, indicating that NK cells are responders to checkpoint blockade [24, 25]. Next, we analyzed the composition of immune landscapes in tumors with high PD-L1 expression from 32 types of cancer. PD-L1 signatures, although not absolutely, did potentially reflect the infiltration of macrophages and T cells, but this was reversely correlated with the expression of lineage markers of NK cells, B cells, or neutrophils in most types of human cancer (Fig. 6a). Reciprocal analyses by categorizing PDAC tumors into high PD-L1 vs. low PD-L1 also revealed a significant difference in NK cell enrichment (Fig. 6b).

Based on the aforementioned findings, we determined whether targeting PKM2 and PD-L1 could enhance the antitumor immunity of NK cells. NK cells isolated from peripheral blood mononuclear cells (PBMCs) were stimulated with interleukin (IL)−2/IL-12 and co-cultured with human PDAC cells. Flow cytometric analysis revealed that the apoptotic killing and inflammatory cytokine secretion of NK cells was significantly inhibited when PDAC cells were pretreated with TGF-β1. However, treatment with the PKM2 targeting compound TEPP-46 and PD-1 checkpoint blockade significantly increased the populations of GzmB^+^ and NKp46^+^ NK cells and decreased tumor cell survival compared with the TGF-β1-treated group (Fig. 6c–g). Additionally, tumor cell apoptosis (Fig. S6a, b) and NK cell cytotoxic activity (Fig. S6c–e) were not affected by any treatment alone. Therefore, NK cell-mediated killing of PDAC cells was enhanced following the blockade of PKM2 nuclear translocation or PD-1/PD-L1 interaction in vitro.

### PKM2 deficiency enhances the antitumor effect of Anti-PD-1 blockade in PDAC in mice with high infiltration of activated NK cells

**Fig. 7:**
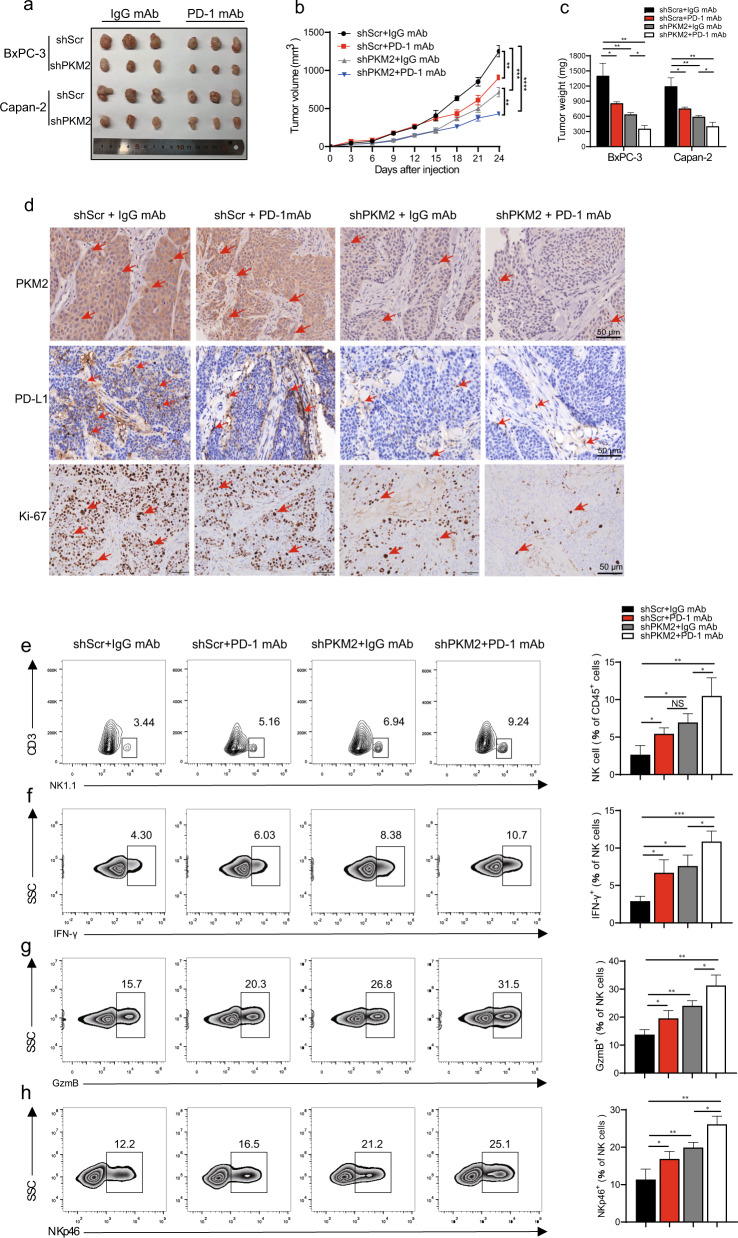
PKM2 knockdown combined with PD-1/PD-L1 blockade significantly restrains tumor growth of PDAC and activates infiltrated NK cells in vivo. BxPC-3/Capan-2 cells with different PKM2 expression levels were inoculated subcutaneously on NOD/SCID mice, and PBS or anti-PD-1 mAb was injected intraperitoneally every 3 days starting on day 9. NOD/SCID mice were euthanized on day 24, and tumor xenografts were isolated and prepared for IHC and flow cytometry. **a** Representative tumor xenografts in each group. **b** Tumor growth curves for each group (*n* = 10). **c** Tumor weights of each group are represented as the means of tumor weights ±SD. **d** Representative images of tumor serial sections stained with PKM2, PD-L1, and Ki-67 by IHC for each group. Red arrowheads showed PD-L1- or PKM2-positive tumor cells. Scale bar, 50 μm. **e** Single-cell suspensions from tumors of each group were stained for flow cytometry. Representative flow cytometry gated on CD3^−^ NK1.1+ cells (*n* = 10). **f**–**h** Representative flow cytometry of (**f**) IFN-γ^+^, (**g**) GzmB^+^, and (**h**) NKp46^+^ NK cells in gated CD3^−^ NK1.1^+^ cells (*n* = 10 per group). Data are shown as mean ± SEM from ten mice per group and are representative of two experiments. Statistical significance was determined by the unpaired Mann–Whitney *U*-test. NS = no significance; ^*^P < 0.05,^**^P < 0.01,^***^P < 0.001, ^****^P < 0.0001.

We have shown that PKM2 knockdown in PDAC cells promoted NK cell infiltration and activation by reducing PD-L1 expression in vivo (Fig. 2f–i). To examine the effects of PKM2 targeting on response to PD-1 blockade and the cytotoxicity of NK cells, we infused NK cells derived from human PBMCs (1 × 10^6^/mice) and/or administered anti-PD-1 (10 mg/kg, 3 of 7 days) to immunodeficient PDAC model where tumor cells were infected with PKM2-shRNA or scramble-shRNA. The effects of anti-PD-1 monotherapy were evaluated and displayed that the combination of PKM2-shRNA and PD-1/PD-L1 blockade had the best antitumor efficacy, resulting in smaller tumor size and lighter tumor weight, followed by PKM2 knockdown and anti-PD-1 therapy alone (Fig. 7a–c). Consistent with these findings, IHC of tumor tissue also showed that the combination therapy markedly decreased the proliferation of tumor cells in PKM2-shRNA animals treated with anti-PD-1 (Fig. 7d). Moreover, flow cytometry of resected PDAC tumors demonstrated a significantly higher percentage of infiltrated NK cells with increasing production of IFN-γ, GzmB, and NKp46 in the PKM2-shRNA + anti-PD-1-treated group than the monotherapy groups (Fig. 7e–h). These observations demonstrated that the synergistic antitumor benefit of combined PKM2 and PD-L1 blockade may provide a potential therapy for treating PDAC.

## Discussion

PDAC is a highly lethal malignancy with few effective therapies [26, 27]. Recently, immunotherapies based on the use of PD-1/PD-L1 antibodies have been postulated as a great promise for cancer treatment [28–30]. However, not all patients with PDAC respond to PD-1/PD-L1 inhibitors, highlighting the need for a better understanding of the regulatory mechanism of PD-L1 [4]. In this study, we bridged the relationship between the glycolytic enzyme PKM2 and PD-L1 expression and illustrated the key role of PKM2 as a prognostic indicator and a therapeutic target in PDAC. Further functional studies have revealed that TGF-β1 secreted by TAMs upregulate PD-L1 levels in PDAC cells by inducing dimeric PKM2 to translocate into the nucleus. In addition, we demonstrated that the interruption of the TGF-β1-mediated PKM2/STAT1-PD-L1 axis could remodel the immune microenvironment by activating NK cells and restrain tumor growth. Therefore, we propose that PKM2 represents an effective therapeutic target for treating PDAC.

Plasma PKM2 is considered a diagnostic biomarker with similar specificity and sensitivity to serum CA19-9 in PDAC [31, 32]. Though researchers have contradictory conclusions about the clinical impacts of tissue PKM2 level on the overall survival of patients with PDAC, most researchers have supported that patients with tumors with high PKM2 expression have worse overall survival than those with tumors with low PKM2 expression [33], which is consistent with our results of TCGA data analysis. PKM2 functions as both a cytosolic metabolic enzyme and a nuclear factor in tumor cells. The balance between metabolic and non-metabolic PKM2 functions, monitored by the dimer/tetramer ratio, appears to be crucial for tumor growth [34]. Studies have shown that relying on its c-terminal nuclear localization signal in response to epidermal growth factor, peroxide, growth hormone inhibitor analog TIT-232, IL-3, ultraviolet radiation, and other factors, PKM2 can transfer from the cytoplasm to the nucleus and function as a protein kinase in the dimer form in the nucleus to affect downstream signaling pathways to promote tumor development [15, 35]. It has been well recognized that PKM2 is capable of translocating to the nucleus and enhancing β-catenin’s transactivation activity, promoting the expression of downstream oncogene cyclin D1 and the progression of cell cycle [36, 37]. Our findings reveal that TAM-derived TGF-β1 is an activator that promotes PKM2 nuclear translocation in PDAC cells and assumes the role of a protein kinase. In patients with PDAC with a high glycolysis activity in tumor sites, which is reflected by the “hot area” in FDG-PET scan, the expression of PKM2 increased. It could be attributed to the overactivation of the glycolysis process [5]. However, in this study, whether the consequence of the activated glycolysis is another factor involved in PKM2 translocation or overall expression still needs to be determined. Future studies should focus on the clinical quantification of glycolysis levels and PKM2 functions in metabolism regulation.

Accumulating evidence suggests that the combination of a few molecules with anti-PD-1/PD-L1 shows an enhanced treatment potential in PDAC, such as TGF-β, CD40, CXCR4, and c-Myc [4, 38, 39]. However, these trials appear to modestly extend survival, and the overall mortality of patients with PDAC remains high [4]. This study provides evidence that concomitant PKM2/PD-L1 pathway inhibition greatly improves their efficacy and further extends survival. In addition, we illustrated that STAT1 alone did not significantly activate PD-L1 transcription, whereas the strengthened PKM2–STAT1 interaction markedly increased the PD-L1-Luc activity. The regulation of PD-L1 by PKM2 in tumor and immune cells is suggested to be associated with several tumors, such as hepatocellular carcinoma and lung cancer [40, 41]. Recent studies have shown that the circadian clock regulates PKM2 expression and further controls the immune checkpoint PD-L1 in a STAT1-dependent manner in sepsis [42]. Our data demonstrated that TGF-β1 secreted by TAMs upregulates the phosphorylation of PKM2 and coactivates STAT1, synergistically promoting PDAC development by inducing PD-L1 transcription to inhibit antitumor immune function.

Immunotherapy targeting the PD-1/PD-L1 axis enhances antitumor immunity mostly by unleashing T cells to attack tumor cells. However, some cancer types exhibit a high incidence of MHC loss or low neoantigen burden [43], which makes it difficult for T cells to recognize tumor cells. Meanwhile, it has been found that certain cancer types with highly expressed PD-L1 but a low expression of MHC I, respond well to PD-1/PD-L1 blockade [44]. Furthermore, despite the loss of MHC I in Hodgkin’s lymphoma, high PD-L1 expression levels predicted a poor prognosis [45]. These findings suggest that in some cancer types, immune responses are inhibited by PD-1/PD-L1 interaction independent of cytotoxic T cells, suggesting that other immune cell types also play a role. NK cells are significant innate lymphocytes against tumor cells via their cytotoxic activity, and in solid tumors, the infiltration of NK cells predicts a good outcome. In PDAC, early and potent NK cell infiltration is positively related to prognosis [46]. Different from T cells, tumor cells can be recognized by NK cells without neoantigens or overexpress self-antigens, and loss of MHC expression even increases their susceptibility to NK cell killing [47]. It has been confirmed that PD-1 is widely expressed on human NK cells in many cancer types, which might compromise NK cell functions [25]. In this study, we established PDAC tumor mouse models subcutaneously and found that the proportion of NK cells infiltrated in TME was significantly increased due to PKM2 knockdown in PDAC cells. Furthermore, NK cells were much more activated in PDAC tumors defective in PKM2 than those in controls. Notably, the combined blockade of PD-1/PD-L1 with PKM2 knockdown could enhance immunity against PDAC by promoting NK cell infiltration into tumors in vivo. These findings suggest that the NK cell is the key effector cell inhibited by PKM2/PD-L1-mediated immunosuppression in PDAC, and the combination of PD-1/PD-L1 blockade with PKM2 depletion has better efficacy than monotherapy, suggesting the potential translational value of this strategy.

## Conclusions

**Fig. 8:**
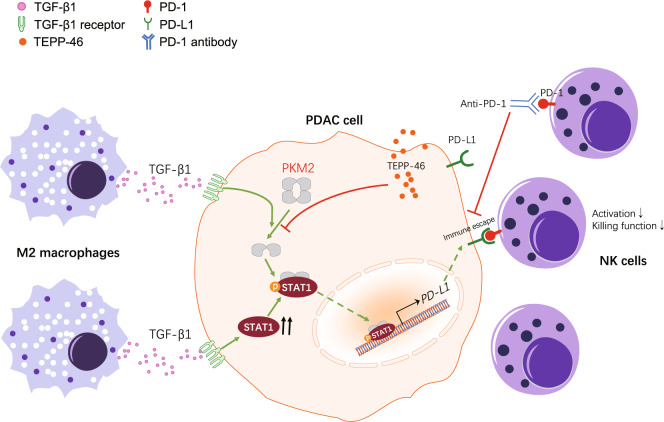
Model for PKM2 regulated TAM-induced PD-L1 expression and immunosuppression in PDAC. In the proposed model, TGF-β1 secreted by TAMs induces the nuclear translocation of PKM2, resulting in PD-L1 overexpression in PDAC cells by the Jak-Stat1 pathway, which leads to NK cell inactivation and dysfunction. Instead, the inhibition of PKM2 or PD-L1 leads to enhanced antitumor immunity in PDAC models.

In conclusion, we have uncovered a significant role of PKM2 in TGF-β1-induced upregulation of PD-L1 in PDAC cells, which further suppresses NK cell-mediated antitumor immunity, and the combination of PD-1/PD-L1 blockade with PKM2 knockdown enhances antitumor immunity in PDAC models, as summarized in our working model (Fig. 8). Our findings are expected to provide a new theoretical basis for immunotherapeutic strategy improvement of PDAC, especially those patients whose tumors have high PKM2 expression levels.

## Supplementary information

Supplementary Files.

Supplementary table 1–3.

## Data Availability

The data supporting the conclusions of this article have been given in this article and its additional files.
