# Supplementary material for: Tumor-associated macrophages promote PD-L1 expression in tumor cells by regulating PKM2 nuclear translocation in pancreatic ductal adenocarcinoma
Source: Oncogene. 2021 Dec 3;41(6):865–77. doi: 10.1038/s41388-021-02133-5 (PMC8816727; doi:10.1038/s41388-021-02133-5)
Supplement: Supplementary file 1 — Supplementary Files. [file 41388_2021_2133_MOESM1_ESM.pdf]

Supplementary Files

Manuscript title: Tumor-associated Macrophages Promote PD-L1 Expression in Tumor Cells by Regulating PKM2 Nuclear Translocation in Pancreatic Ductal Adenocarcinoma

Authors: Qing Xia[1#], Jing Jia[2#], Chupeng Hu[3,4,5#], Jinying Lu[3,4,5], Jiajin Li[6], Haiyan Xu[7], Jianchen Fang[8], Dongju Feng[4], Liwei Wang[7*], Yun Chen[3,4,5*]

Supplementary Materials and Methods

Cell culture

BxPC-3 and Capan-2 cells were cultured in complete 1640 medium (Cat. No. C22400500BT, Gibco), and HPNE cells were cultured in complete HTERT-HPNE cell specific medium (Cat. No. iCell-h102-001b, iCell Bioscience Inc, Shanghai), supplemented with 10% fetal bovine serum (Cat. No. 16000-044, Gibco), 100 U/mL penicillin, 100 µg/mL streptomycin (Cat. No. 15 140–22, Gibco), and 2 mM L-GlutaMax (Cat. No. 35050061, Gibco) at 37°C in a humidified 5% CO2 atmosphere. Fludarabine was purchased from Seleckchem, TEPP-46 was from Biovision, rh-TGF-β1 was from Peprotech, and anti-PD-1 monoclonal antibody (Nivolumab, OPDIVO) was from Bristol Myers Squibb.

Reagents

Antibodies against PKM2, caspase-3, histone H3, PD-L1, STAT1, p-STAT1, Smad2, p-Smad2, Smad-3, and p-Smad3 were obtained from Cell Signaling Technology. VENTANA PD-L1 (SP263) Assay for IHC was from Roche Diagnostics. Anti-rabbit Ki-67, Anti-rabbit CK19, anti-rabbit CD68, anti-rabbit CD163, and anti-rabbit CD206 were obtained from Abcam. Antibodies against β-actin (MA5-15739, mouse) were purchased from Thermo Fisher Inc. Additional details are provided in Supplementary Material (Supplementary Table 2).

Animal

Male 6–8-week-old nude mice and NOD/SCID mice were purchased from the Model Animal Research Center of Nanjing University. Mice experiments were conducted according to the National Institutes of Health Guidelines for the Care and Use of Laboratory Animals. The study procedures were performed with the approval of the Institutional Animal Care and Use Committee of Animal Core Facility of Nanjing Medical University.

Tumor model experiment

For xenograft mice models, BxPC-3 and Capan-2 cell lines were infected with the scrambled or PKM2 knockdown lentivirus, nude mice were injected subcutaneously with 3×10^6 scrambled or sh-PKM2 BxPC-3/Capan-2 cells in 100-μL PBS on the right axilla, respectively (n = 10 per group). Tumor size was measured once every other day using a vernier caliper. Tumor volume was calculated based on the two perpendicular measurements and using the

following formula: volume = (length × width^2)/2. When the tumor volumes reached the endpoint criteria (diameter, 10–15 mm), all mice were euthanized, and the tumors were collected for further fluorescence-activated cell sorting (FACS) and IHC analysis.

Human NK cell sorting and adoptive transfer experiments

A FACS Aria cell sorter (BD Biosciences) was used to purify NK cells (CD3-CD56+) from PBMCs. The purity of the sorted NK cell populations was more than 95%, as verified by post-sort flow cytometry. For the infusion of human NK cell experiment, after receiving subcutaneous injections of 3 × 10^6 scrambled or sh-PKM2 BxPC-3/Capan-2 cells in 100-μL PBS on the right axilla, respectively, mice were treated with anti-PD-1 mbA or IgG mbA (10 mg/kg, 3 of 7 days). After being irradiated to destroy the immune system, NOD/SCID mice were infused with human NK cells (1 × 10^6/mice) on day 5. Ten mice were included in each group. Tumor volumes were assessed as the aforementioned tumor model experiment. When the tumor volumes reached the endpoint criteria (diameter, 10–15 mm), all mice were euthanized, and the tumors were collected for further FACS and IHC analysis.

IHC analysis

Tumor tissues were fixed in 4% paraformaldehyde for at least 48 h and then embedded in paraffin. The samples were sectioned at 5-μm thickness. These sections were rehydrated and then boiled in 10-mM citric acid buffer and pH 6.0 at 95°C for antigen retrieval, and endogenous peroxidases were quenched

with 3% hydrogen peroxide. The slides were blocked with 5% goat serum for 1 h at room temperature and then incubated with an optimal dilution of a primary rabbit antibody overnight at 4°C. After three 5-min washes with PBS, the samples were stained with fluorescent secondary antibodies for 1 h at room temperature, followed by DAB staining for nuclei visualization. Images were acquired and analyzed using a Zeiss fluorescence microscope with the Axiovision image analysis software.

qRT-PCR

Following the manufacturer's instructions, total RNAs were extracted using TRIzol reagent (Invitrogen, USA). RNA samples were reverse-transcribed into cDNA using HiScript®Q Select RT SuperMix. qRT-PCR was performed using an AceQ® qPCR SYBR Green Master Mix (High ROX Premixed) Kit (Thermo Fisher Scientific, USA) in a 7500 Fast Real-Time PCR System (Applied Biosystems, USA). The results were standardized to control β-actin values. The primers used in these assays are presented in Supplementary Table 3.

Short hairpins, plasmids, and lentiviral transduction and infection

PKM2-specific shRNAs originate from the "MISSIONshRNA Library" designed and developed by the TRCat the Broad Institute of MIT and Harvard. One PKM2 hairpin (NM_182471.1-1706s1c1- #) that showed high-efficacy knockdown was selected. High-titer virus-containing supernatants of HEK293FT cells after transient co-transfection of lentiviral vectors with pMD2.G and psPAX2 packaging vectors were used for lentiviral-mediated

transduction of cancer cells.

Western blotting

Cells were collected and washed twice using cold PBS. Cell extracts were collected and quantified using the BCA Protein Assay Kit (Thermo Fisher Scientific, USA). Equal amounts of the proteins were electrophoresed on SDS polyacrylamide gels for separation, transferred to PVDF membranes (Millipore, USA), and blocked with 5% non-fat dry milk in PBS (PBST) at room temperature for 1 h. The membranes were incubated with primary antibodies at 4 ℃ overnight and then incubated with an alkaline phosphatase-conjugated goat anti-mouse or anti-rabbit IgG secondary antibody (Jackson, 1:5000 dilution) for 1 h at room temperature. An enhanced chemiluminescence detection system was used to detect the immunoreactive protein bands according to the manufacturer's instructions. Each experiment was repeated at least three times.

Apoptosis assays

Adherent and nonadherent cells were harvested and washed three times with cold PBS. Cell apoptosis was determined by staining with PE Annexin V and 7-AAD. Cells that were considered viable were PE Annexin V- and 7-AAD-negative; cells that were in early apoptosis were PE Annexin V-positive and 7-AAD-negative; and cells that are in late apoptosis or already dead are both PE Annexin V-positive and 7-AAD-positive. The percentage of apoptotic cells was analyzed using the FlowJo software.

Flow cytometry analysis

The single cells prepared from the collected tumor of mice or the treated NK cells isolated from PBMCs were harvested and labeled with indicated antibodies. In detail, the subcutaneous tumors were digested and homogenized by crushing using the head of a 1-mL syringe in a Petri dish, followed by straining through a 70-μm nylon filter to obtain a single-cell suspension. For intracellular cytokine staining, cells were incubated for 4–5 h with 50 ng/mL PMA, 750 ng/mL ionomycin, and 10 μg/mL BFA (all from MultiSciences Biotech) in a cell culture incubator at 37°C. Cells were resuspended in fixation/permeabilization solution, and intracellular cytokine staining was performed (Thermo Fisher Scientific, USA). Antibodies for flow cytometry staining are listed in Supplementary Table 2. Data were collected using a CytoFLEX flow cytometer (Beckman Coulter, USA) and analyzed using the FlowJo software (Tree Star, Ashland, OR, USA).

Immunofluorescence

BxPC-3 cells in a confocal dish were fixed with 4% paraformaldehyde at room temperature for 30 min. The coverslips were washed three times with PBS, and the fixed cells were treated with 0.3% Triton X-100 at room temperature for 10 min to permeabilize the plasma membrane and washed three times with PBS. The cells in the confocal dish were blocked overnight at 4°C with 1% w/v bovine serum albumin (BSA) in PBS. Primary antibodies were diluted in 1% w/v BSA. Following staining, the coverslips were washed three times with PBS.

Secondary antibodies were diluted with PBS containing 1% w/v BSA. Following staining, the coverslips were washed three times with PBS, mounted in Prolong Gold with 4′,6-diamidino-2-phenylindole dihydrochloride reagent. The immunofluorescence was imaged using a Zeiss LSM 800 confocal microscope, and analyzed using the line profile tool in the LSM 800 META ZEN software package (Carl Zeiss). Multiplex immunofluorescence was performed using three kits (B40922/B40923/B40926; Invitrogen, CA, USA) according to the reagent manufacturer’s instructions. Briefly, slides were sequentially stained with specific antibodies, positively stained regions of all images were extracted, aligned, overlaid, and segmented using Image-Pro plus 6.0.

Polarization of macrophages

Human PBMCs were treated with M-CSF (Peprotech) for differentiation into macrophages (M0) before polarization. For skewing to an M1 phenotype, LPS (Sigma) and IFN-γ (Peprotech) were added to culture. For skewing to an M2 phenotype,IL-4 (Peprotech) and IL-13 (Peprotech) were added to culture.

Macrophage and tumor cell co-culture

The co-cultivation of macrophages and tumor cells was performed using the noncontact co-culture transwell system. Then, 2×10^5 polarized macrophages were seeded in 1.0-μm pore inserts with a fresh medium for 24 h. The inserts containing M1 or M2 polarized macrophages were transferred to 6-well cell culture plates seeded with BxPC-3 or Capan-2 cells (1×10^5 cells per well) in advance and co-cultured for another 48 h. Then, the inserts were discarded,

and the BxPC-3 or Capan-2 cells were washed with cold PBS three times, and the supernatant medium was collected for further antibody array analysis.

Chemical cross-linking assay

BxPC-3 or Capan-2 cells were lysed with sodium phosphate buffer (pH 7.3) containing 0.5% Triton X-100 and 1 × protease inhibitors (Thermo Fisher Scientific, USA) for 30 min at 4°C. Crude cell lysates were clarified by centrifugation at top speed (16,000 × g) for 30 min at 4°C. To perform cross-linking reactions, the supernatants were treated with 0.01% or glutaraldehyde for various periods (0–5 min) at 37°C. The reaction was terminated by adding 1-M Tris buffer to a final concentration of 50-mM Tris•Cl (pH 8.0). The samples were then separated by 5%–15% SDS/PAGE and analyzed by Western blotting with anti-PKM2 antibody as indicated.

Subcellular fractionation

The fractionation of nuclear and cytosolic extracts was performed by using the PARIS™ Kit (Thermo Fisher Scientific, USA) according to the manufacturer’s instructions.

Antibody array

RayBio® Human Angiogenesis Antibody Array 1 (G-Series, RayBiotech), allowing simultaneous detection of 20 angiogenic-related proteins, was used to assess secretion profiles in conditioned media of BxPC-3 cells according to the manufacturer's instructions. Arrays were visualized using the ImageQuant LAS4000 software (GE Healthcare) and analyzed using ImageJ (National

Institutes of Health).

RNA sequencing

We treated 1 × 10^6 BxPC-3 cells with 20-ng/mL Recombinant Human TGF-β1 (Peprotech, USA) or with 10-mM Citric Acid (Mock treatment) for 12 h. Then, total RNA samples were collected using TRIzol reagent for whole-genome transcriptome profiling by RNA sequencing. The RNA sequencing and bioinformatics data analysis were performed by Shanghai Biotechnology Corporation. In brief, total RNA was isolated using the RNeasy Micro Kit (Qiagen, Hilden, Germany) and purified using the RNAClean XP Kit (Beckman Coulter, Inc., CA, USA). Paired-end libraries were then synthesized using the TruSeq® RNA Sample Preparation Kit (Illumina, USA), following the TruSeq® RNA Sample Preparation Guide. The purified libraries were sequenced on the Illumina HiSeq X-ten (Illumina, USA). For data processing, we used Hisat2 (version 2.0.4) to map the cleaned reads to the human GRCh38 reference genome with two mismatches. After genome mapping, Stringtie (version:1.3.0) was run with a reference annotation to generate fragments per kilobase of transcript per million mapped read values for known gene models. *P*-values and fold changes were used to define the threshold of significance.

IP

Following the manufacturer's instructions, IP/Co-IP assay was performed using the Pierce™ Classic Magnetic IP/Co-IP Kit (Thermo Fisher Scientific, USA). In brief, cells were lysed in an IP Lysis/Wash Buffer. The cell lysate was

incubated with the chosen antibody for IP overnight at 4ºC. Then, the protein complexes were collected by incubation with 25-μL Pierce Protein A/G Magnetic Beads for 1 h at room temperature. The collected protein complexes were washed twice with IP Lysis/Wash Buffer and once with purified water and then analyzed by Western blotting.

Ch-IP

Ch-IP was performed according to the Magna ChIP™ Protocol. Chromatin from treated BxPC-3 cells was incubated overnight with either anti-PKM2 or STAT1 as the immunoprecipitating antibody (standard protocol). Purified DNA was then analyzed by qPCR. Fold enrichment, the ratio of PKM2 or STAT1 signal to IgG signal, was calculated by a standard curve of input DNA dilutions. PKM2 and STAT1 antibodies were acquired from Cell Signaling Technology. The qPCR primers were used as follows: Pd-l1 promoter: 5'-ATGAAACTTGTTGTACATGTGTGTG-3' and 5'-AGCAATTTTGGTGACTGTAAGTTTG-3', 5'-CTATTGCAATTTTATTGGGGCC-3' and 5'-GCATTGGTTATGACATTTGCTTCC-3', 5'-GGGTCTGCTGCTGACTTTTTATATG-3' and 5'-CAACATCTGAACGCACCTTGATT-3', and 5'-GTGGATTTGCTTTAATCTTCGAAAC-3' and 5'-TCAGTTTAGGTATCTAGTGTTGGTG-3'.

Promoter reporter assay

Putative STAT1-binding domains in PD-L1 promoters were amplified and inserted into pGL3-based luciferase reporter plasmids, followed by transfection into 293T cells. Then, 5 million cells were collected 48 h after transfection, and luciferase activity was detected using a Dual-Luciferase Reporter Assay System (Promega) according to the manufacturer’s protocol.

Biochemical assays

The glucose uptake level was determined using a Glucose Assay Kit (Sigma, USA), as described by the manufacture’s protocol. The lactate concentration of the cell supernatant or the tumor tissues were determined using a Lactate Testing Kit (Nanjing Jiancheng Bioengineering Institute, Nanjing, China), according to the manufacture’s protocol.

EdU assay

EdU experiments were performed using a Cell-Light EdU Apollo567 In Vitro Kit (Cat.C10310-1, Ruibo, Guangzhou, China) according to the manufacturer’s instructions.

Statistics

Statistical analysis was performed with the Prism 7.0 software (GraphPad Software, Inc., La Jolla, CA, USA). Statistical significance was assessed by unpaired Student t-test or Mann–Whitney U-test. P-values of less than 0.05 were used to denote statistical significance. The quantitative analysis of immunohistology staining was performed using the Image J software (National Institute of Health, Bethesda, MD, USA) using the Colour Deconvolution

plugin.

Supplementary Figures

Figure S1

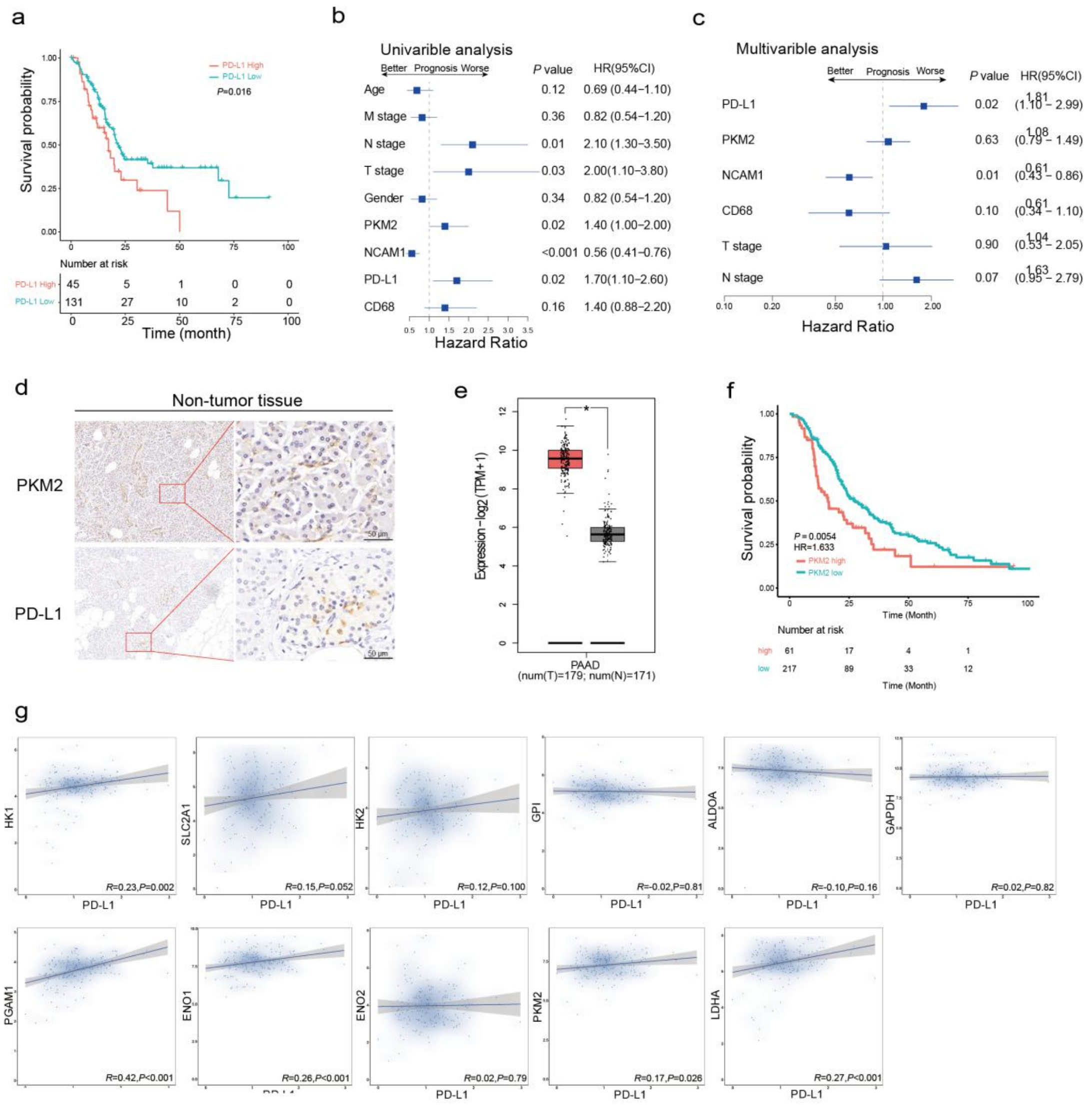

Figure S1 PD-L1 expression was correlated to poor prognosis in patients with PDAC. (a) Kaplan-Meier curves for overall survival in PDAC patients with high or low PD-L1 expression based on TCGA data set. **(b-c)** Univariate **(b)** and multivariate **(c)** regression analyses of factors associated with recurrence. Cox proportional hazards regression model. **(d)** Representative immunostaining of PD-L1 and PKM2 from paired non-tumor adjacent tissue

samples from the PDAC patients. **(e)** The mRNA levels of PKM2 in PDAC tumor tissues and non-tumor pancreatic tissues from the TCGA and GTEx datasets. T = tumor, N = non-tumor. **(f)** The overall survival of patients with PDAC based on PKM2 expression according to the E-MTAB-6134 dataset. Analysis was performed using Kaplan–Meier estimates and two-sided log-rank tests. **(g)** Correlation between glucose metabolism and PD-L1 expression in PDAC patients based on TCGA data set. The relative gene of glucose metabolism included SLC2A1, HK1,HK2, GPI, ALDOA, PGAM1, ENO1,ENO2, PKM2, LDHA. P values were indicated in each plot.

Figure S2

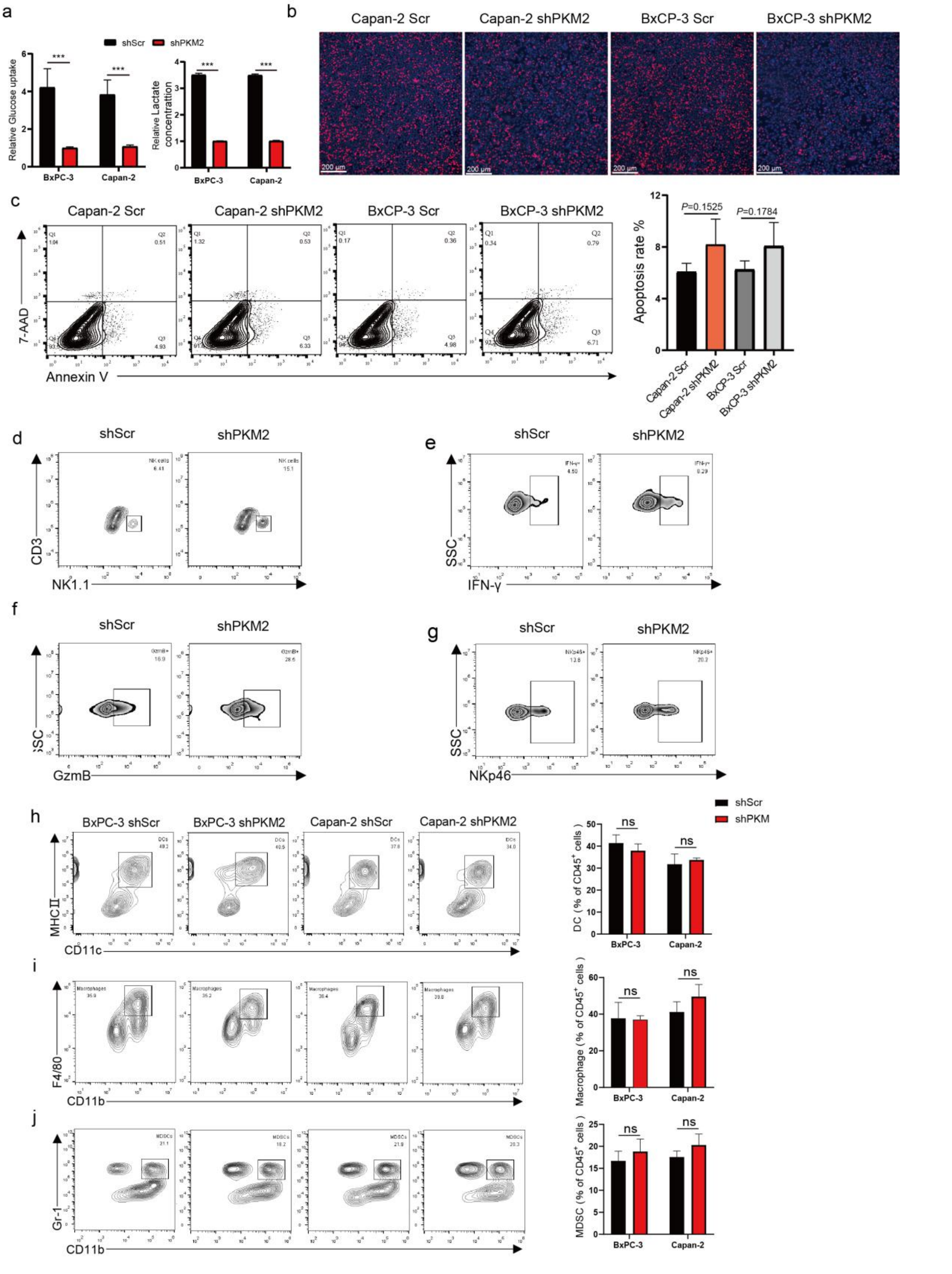

Figure S2 PKM2 knockdown PDAC tumors increased infiltration of NK cells, Related to Figure 2

(a) The effects of PKM2 knockdown on the glucose uptake and lactate levels in BxPC-3/Capan-2 cells. **(b)** EdU assays of Capan-2 and BxPC-3 cells infected

with a PKM2-specific or non-targeting shRNA. **(c)** Capan-2 and BxPC-3 cells infected with a PKM2-specific or non-targeting shRNA were harvested for apoptosis analysis by flow cytometry. **(d-j)** Single cell suspensions from BxPC-3/Capan-2 tumors with different expression levels of PKM2 isolated from NOD/SCID mice on day 24 were stained for flow cytometry. **(d-g)** flow cytometry plots showed IFN-γ^+, $GzmB^+$ and $NKp46^+$ NK cell from Capan-2 tumor with different expression levels of PKM2 (n=10 per group). **(h-j)** Representative flow cytometry plots (left) and percentages (right) of tumor-infiltrating leukocyte subsets including DC cells **(h)**, macrophages **(i)**, MDSC **(j)** in gated $CD45^+$ cells were showed. (n=10 per group) Data are shown as mean ± SEM from 10 mice per group and are representative of two separate experiments. Statistical significance was determined by Mann whitney U test. NS=no significance.

Figure S3

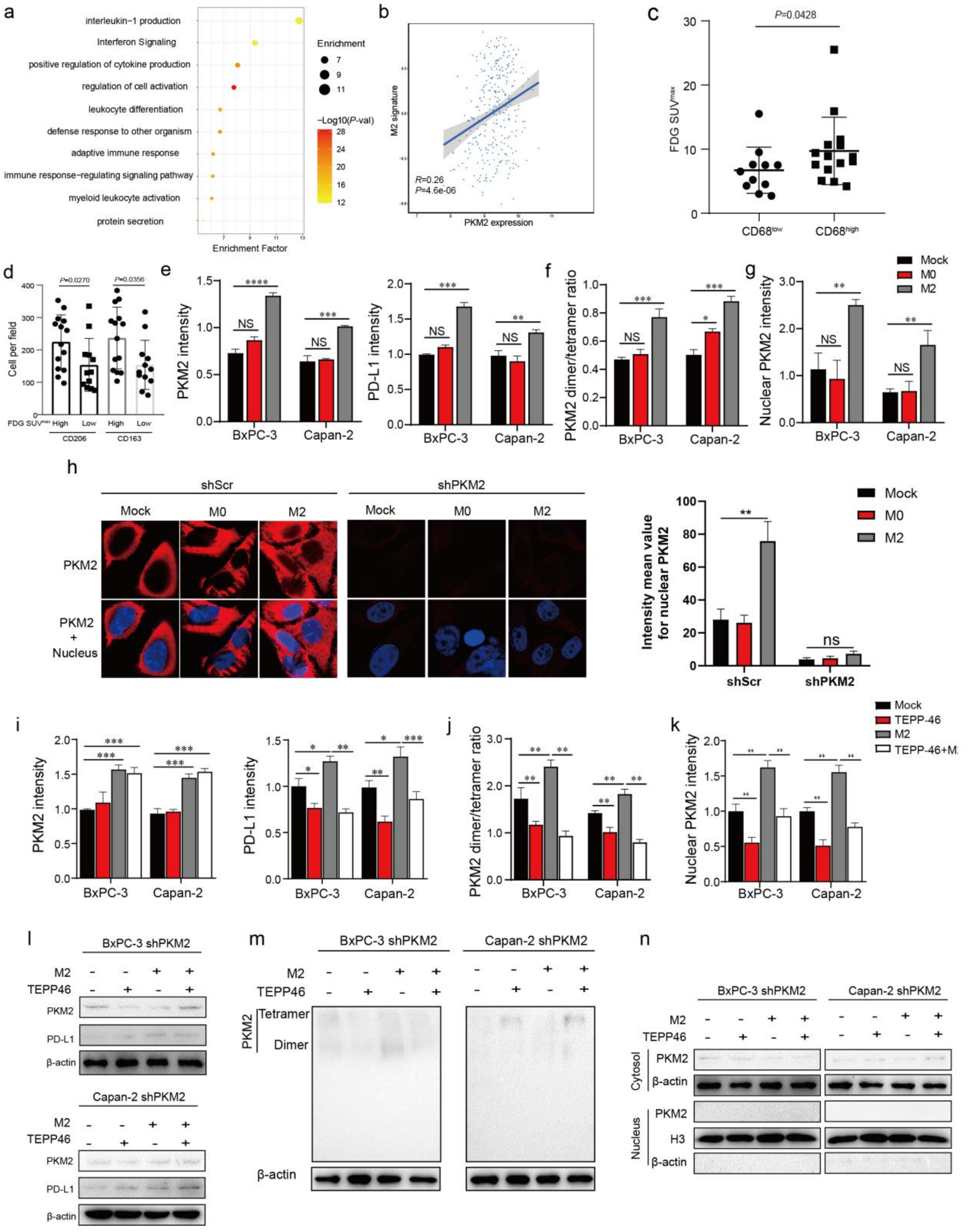

Figure S3 M2 macrophages upregulate PD-L1 expression of tumor cells, Related to Figure 3. (a) Top 10 biological processes (GO terms) enriched in 270 genes correlated with PD-L1 expression in PDAC tissues from TCGA data set (R > 0.5; *P* < 0.0001). **(b)** Correlation between M2 macrophage signature and PKM2 was calculated in PDAC from the E-MTAB-6134 dataset. **(c)** FDG

SUVmax values for PDAC tumors with low or high CD68+ macrophages (n =26). **(d)** Correlations of CD206+ macrophage and CD163+ macrophage densities with PD-L1 expression in PDAC tissues (n = 26). **(e)** The integrated density value of PKM2 (left) and PD-L1 (right) was quantified in Figure 3f. **(f)** PKM2 dimer/tetramer ratio was quantified in Figure 3g. **(g)** Nuclear PKM2 intensity value was quantified in Figure 3h. **(h)** Subcellular localization of PKM2 in BxPC-3/Capan-2 cells infected with a PKM2-specific or non-targeting shRNA co-cultured with M0/M2 macrophages. Cells were immunostained with anti-PKM2 (PKM2, red). The nucleus was marked with 4′,6-diamidino-2-phenylindole dihydrochloride (blue). **(i)** The integrated density value of PKM2 (left) and PD-L1 (right) was quantified Figure 3j. **(j)** PKM2 dimer/tetramer ratio was quantified in Figure 3K. **(k)** Nuclear PKM2 intensity value was quantified in Figure 3l. **(l)** BxPC-3/Capan-2 cells infected with a PKM2-specific shRNA were pretreated with TEPP-46 (10 μM) for 12 h or/and then co-cultured with M2 polarized macrophages (tumor cell:macrophage = 1:2) for 48 h. Then, PKM2 and PD-L1 protein expression levels from whole cell lysate of BxPC-3/Capan-2 shPKM2 cells were determined by Western blotting. **(m)** BxPC-3/Capan-2 shPKM2 cells pretreated with TEPP-46 or/and co-cultured with M2 macrophages were cross-linked by glutaraldehyde first and then subjected to Western blotting. **(n)** Nuclear and cytosolic lysates were prepared from BxPC-3/Capan-2 shPKM2 cells pretreated with TEPP-46 or/and co-cultured with M2 macrophages,

followed by Western blotting. All intensity values were caculated using ImageJ software and was normalized to β-actin. Data were shown as mean ± SEM from three independent experiments. Statistical significance was determined by Mann whitney U test; *P<0.05, **P<0.01,***P<0.001, NS=no significance.

Figure S4

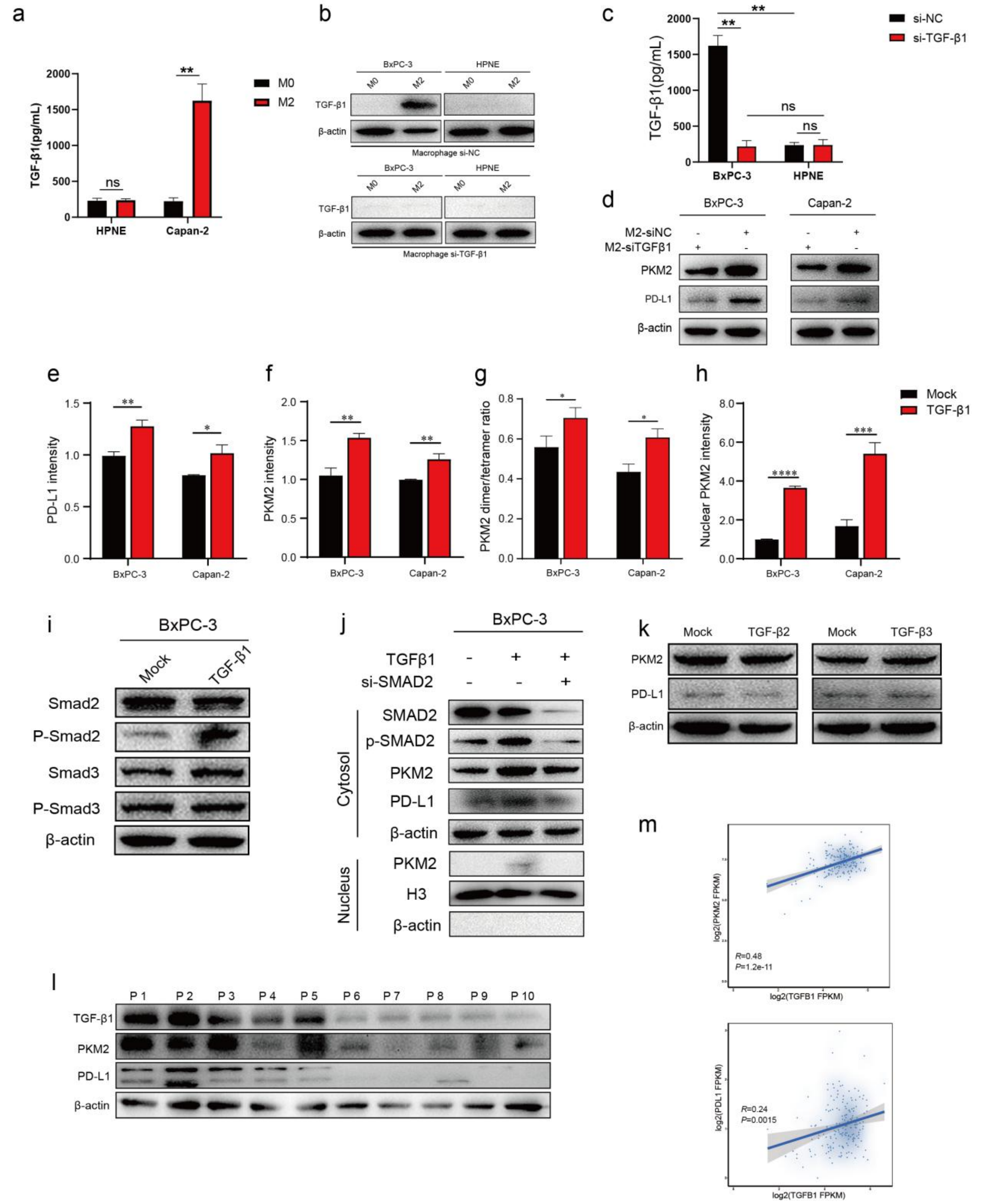

Figure S4 TGF-β1 secreted by M2 macrophages promotes nuclear translocation of PKM2 in PDAC cells, related to Figure 4. (a) HPNE cells or Capan-2 cells were co-cultured with M0 or M2 macrophages. TGF-β1 levels in the supernatant were detected by ELISA. **(b)** The macrophages transfected with siRNAs specific for TGF-β1 or non-targeting control were co-cultured with

BxPC-3/HPNE cells. The protein levels of TGF-β1 in the macrophages were detected by Western blotting. **(c)** The M2 macrophages transfected with siRNAs specific for TGF-β1 or non-targeting control were co-cultured with BxPC-3/HPNE cells. TGF-β1 levels in the supernatant were detected by ELISA. **(d)** The M2 macrophages transfected with siRNAs specific for TGF-β1 or non-targeting control were co-cultured with BxPC-3/Capan-2 cells. The protein levels of PKM2 and PD-L1 in the tumor cells were detected by Western blotting. **(e-f)** The integrated density value of PKM2 **(e)** and PD-L1 **(f)** was quantified in Figure 4d. **(g)** PKM2 dimer/tetramer ratio was quantified in Figure 4e. **(h)** Nuclear PKM2 intensity value was quantified in Figure 4f. **(i)** BxPC-3 cells were treated with or without TGF-β1 (20 ng/mL), and the Smad2, p-Smad2, Smad3 and p-Smad3 protein expression levels in the tumor cells were determined by Western blotting. **(j)** BxPC-3 cells were transfected with Smad2 siRNA, treated with TGF-β1 (20 ng/mL), and then were analyzed by Western blotting for the protein level of PD-L1. **(k)** BxPC-3/Capan-2 cells were treated with or without TGF-β2/TGF-β3 (20 ng/mL), and the PKM2 and PD-L1 protein expression levels in the tumor cells were determined by Western blotting. **(l)** TGF-β1, PKM2 and PD-L1 protein expression levels in PDAC tissues were detected by Western blotting. **(m)** The correlation analysis between PKM2/PD-L1 and TGF-β1 in the TCGA dataset. All intensity values were caculated using ImageJ software and was normalized to β-actin. Data were shown as mean ± SEM from three independent experiments. Statistical

significance was determined by Mann whitney U test; NS=no significance, *P<0.05,**P<0.01,***P<0.001.

Figure S5

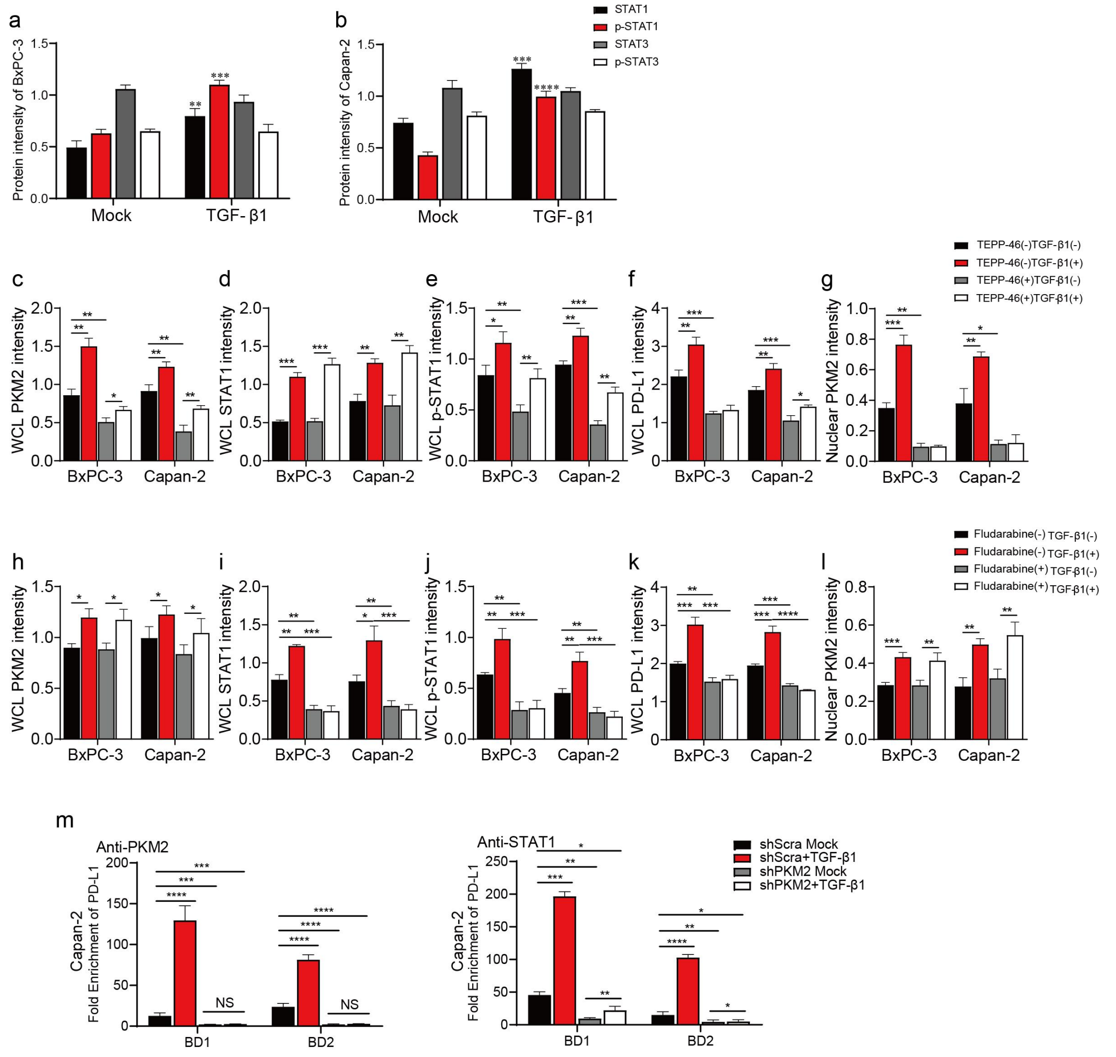

Figure S5 The expression levels of STAT1 and p-STAT1 are significantly upregulated by TGF-β1 stimulation. (a-b) The integrated density value of STAT1/3 and p-STAT1/3 in BxPC-3 (a) and Capan-2 cells was quantified in Figure 5c. **(c-g)**The density value of WCL PKM2**(c)**, STAT1 **(d)**, p-STAT1 **(e)**, PD-L1 **(f)**, and Nuclear PKM2 **(g)** in BxPC-3and Capan-2 cells was quantified in Figure 5G. **(h-l)** The density value of WCL PKM2 **(h)**, STAT1 **(i)**, p-STAT1 **(j)**, PD-L1 **(k)**, and Nuclear PKM2 **(l)**in BxPC-3and Capan-2 cells was quantified in Figure 5H. **(m)** Binding of STAT1 or PKM2 to the two BDs (binding domains) within *PD-L1*promoter was analyzed using ChIP-qPCR in Capan-2 cells

following treatment with TGF-β1 (20ng/ml) for 12 hours.Data were shown as mean ± SEM from three independent experiments; Statistical significance was determined by Mann whitney U test; *$P<0.05$,**$P<0.01$,***$P<0.001$.

Figure S6

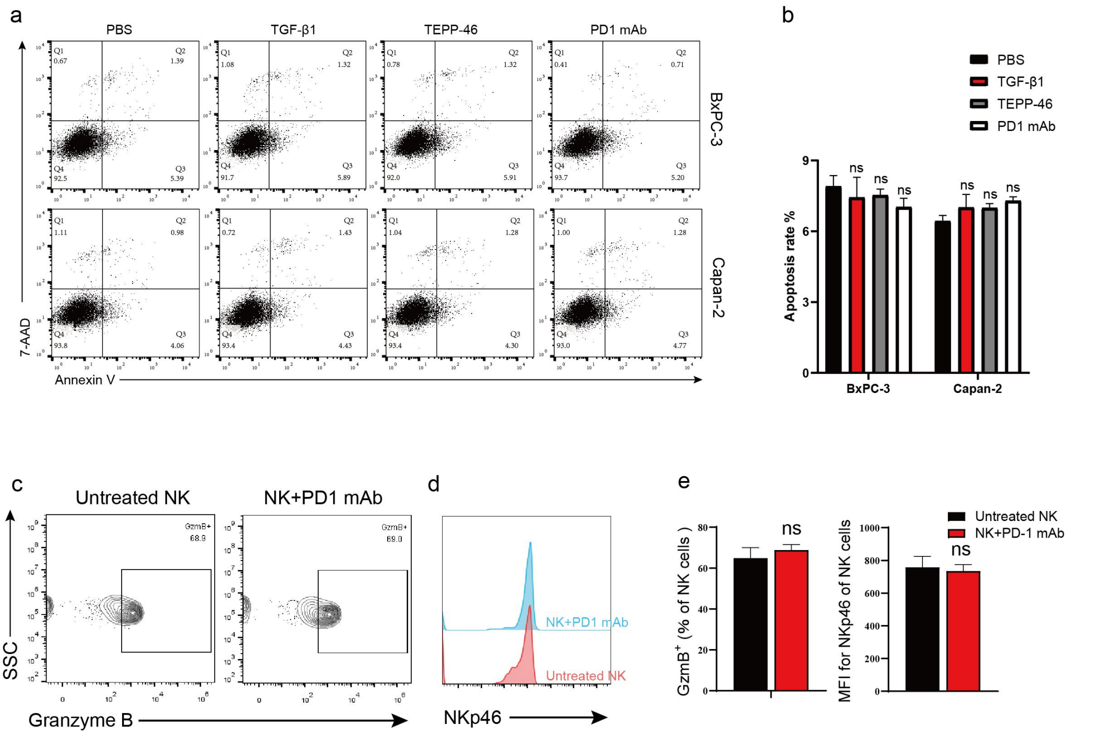

Figure S6 tumor cell apoptosis and NK cell cytotoxic activity were not impacted by any of the treatments alone. (a-b) BxPC-3/Capan-2 cells were treated alone with PBS, TGF-β1(20ng/ml), TEPP-46 (10μM) and anti-PD-1 monoclonal antibody (20μg/ml), respectively for 12 hours. Then conditioned tumor cells were harvested and washed for apoptosis analysis by flow cytometry **(a)**. Apoptosis rate of BxPC-3/Capan-2 cells was showed in **(b)**. **(c-e)** NK cells were isolated from PBMC of healthy volunteers using human NK cell isolation kit (Miltenyi Biotec). The isolated NK cells were treated with anti-PD-1 monoclonal antibody (20μg/ml) for 12 hours. Representative flow cytometry plots **(c-d)** and the percentages **(e)** of GzmB^{+} and NKp46^{+} NK cell were showed. Data are shown as mean ± SEM are representative of two separate experiments. Statistical significance was determined by Mann whitney U test. NS=no significance.
