# Supplementary material for: Tumor-associated macrophages promote PD-L1 expression in tumor cells by regulating PKM2 nuclear translocation in pancreatic ductal adenocarcinoma
Source: Oncogene. 2021 Dec 3;41(6):865–77. doi: 10.1038/s41388-021-02133-5 (PMC8816727; doi:10.1038/s41388-021-02133-5)
Supplement: Supplementary file 2 — Supplementary table 1–3. [file 41388_2021_2133_MOESM2_ESM.docx]

**Table S1** Clinical and pathological characteristics of PDAC patients

| **Characteristics** | **FDG SUVmax** | | | **P value** |
| --- | --- | --- | --- | --- |
|  | **Low(n=13)** | **High(n=13)** | |  |
| Age |  | |  |  |
| ≤65 years | 9（34.62%） | | 7（26.92%） | 0.688 |
| ＞65 years | 4（15.38%） | | 6（23.08%） |  |
| Gender |  |  | |  |
| Male | 8（30.77%） | | 7（26.92%） | ＞0.999 |
| Female | 5（19.23%） | | 6（23.08%） |  |
| T stage |  | |  |  |
| T1  T2  T3  Tx  N stage  N0  N1  N2  Nx  TNM stage  Ⅰ  Ⅱ  Ⅲ  Ⅳ  Unclassified | 4（15.38%）  4（15.38%）  4（15.38%）  1（3.85%）  6（23.08%）  4（15.38%）  2（7.69%）  1（3.85%）  3（11.54%）  5（19.23%）  2（7.69%）  3（11.54%）  0（0.00%） | | 0（0.00%）  6（23.08%）  6（23.08%）  1（3.85%）  4（15.38%）  8（30.77%）  1（3.85%）  0（0.00%）  4（15.38%）  7（26.92%）  1（3.85%）  0（0.00%）  1（3.85%） | 0.164  0.626  0.145 |
| PD-L1 |  | |  |  |
| Low | 8（30.77%） | | 3（11.54%） | 0.047* |
| High | 5（19.23%） | | 10（38.46%） |  |
| PKM2  Low  High  CD68  Low  High  CD206  Low  High  CD163  Low  High | 10（38.46%）  3（11.54%）  8（30.77%）  5（19.23%）  9（34.62%）  4（15.38%）  8（30.77%）  5（19.23%） | | 4（15.38%）  9（34.62%）  3（11.54%）  10（38.46%）  4（15.38%）  9（34.62%）  7（26.92%）  6（23.08%） | 0.018*  0.047*  0.049*  0.069* |

NOTE: PD-L1/PKM2/CD68/CD206/CD163 - low is lower than or equal to median expression; high is higher than median expression. All values in boldface are significant at a level of *P* < 0.05.

**Table S2** Key resources table

| REAGENT or RESOURCE | SOURCE | IDENTIFIER |
| --- | --- | --- |
| Anti-rabbit PKM2 | Cell Signaling Technology | Cat# 4053 |
| Anti-rabbit Caspase-3 | Cell Signaling Technology | Cat# 9662 |
| Anti-rabbit Histone H3 | Cell Signaling Technology | Cat# 4499 |
| Anti-rabbit PD-L1 | Cell Signaling Technology | Cat# 13684 |
| Anti-rabbit Stat1 | Cell Signaling Technology | Cat# 14994 |
| Anti-rabbit p-Stat1 | Cell Signaling Technology | Cat# 9177 |
| Anti-rabbit Ki-67 | Abcam | Cat# ab15580 |
| Anti-mouse β-actin | ThermoFisher | Cat# MA5-15739 |
| Anti-rabbit CD68 | Abcam | Cat# ab213363 |
| Anti-rabbit CD163 | Abcam | Cat# ab182422 |
| Anti-rabbit CD206 | Abcam | Cat# ab64693 |
| Anti-Mouse CD3-FITC | BD Pharmingen™ | Cat# 554832 |
| Anti-Mouse NK-1.1-PE | BD Pharmingen™ | Cat# 557391 |
| Anti-Mouse IFN-γ-APC | BD Pharmingen™ | Cat# 562018 |
| Anti-Human/Mouse GranzymeB-BV421 | BD Pharmingen™ | Cat# 515405 |
| Anti-Mouse NKp46-Alexa Fluor® 700 | BD Pharmingen™ | Cat# 560757 |
| [Anti-Mouse CD45](https://www.bdbiosciences.com/cn/applications/research/stem-cell-research/cancer-research/mouse/fitc-rat-anti-mouse-cd45-30-f11/p/553080)-FITC | BD Pharmingen™ | Cat#553080 |
| Anti-Mouse CD11c-PE | BD Pharmingen™ | Cat#553802 |
| [anti-mouse F4/80](http://bioec.cn/r/123126-BLG)-PerCP | Biolegend | Cat#123126 |
| anti-mouse/human CD11b-APC | Biolegend | Cat#101212 |
| [anti-Mouse LY-6G/ LY-6C](https://www.bdbiosciences.com/cn/reagents/research/antibodies-buffers/immunology-reagents/anti-mouse-antibodies/cell-surface-antigens/v450-rat-anti-mouse-ly-6g-and-ly-6c-rb6-8c5/p/560453)-V450 | BD Pharmingen™ | Cat#560453 |
| Anti-mouse NKp46-PE | Biolegend | Cat#137630 |
| anti-human NKp46-BV421 | BioLegend | Cat#331913 |
| anti-human CD56-APC | BioLegend | Cat#318309 |
| anti-human CD3-PE | BioLegend | Cat#300307 |
| [TEPP-46](http://bioec.cn/r/B2164-5-BV) | Biovision | Cat#B2164-5 |
| [Human TGF-β1](http://bioec.cn/r/AF-100-21C-2UG-PPT) | PeproTech | Cat#AF-100-21C-10UG |
| Anti-PD-1 [5C4.B8 (Nivolumab)] | Bristol Myers Squibb | Cat#Ab00791-13.12 |
| Anti-CK19 | Abcam | Cat#ab76539 |
| TGFb1 inhibitor | MCE | Cat#HY-P0118 |
| Smad2 (D43B4) | CST | Cat#5339T |
| Phospho-Smad2 (Ser465/467) | CST | Cat#3108T |
| Smad3 (C67H9) | CST | Cat#9523T |
| Phospho-Smad3 (Ser423/425) | CST | Cat#9520T |
| HiPerFect Transfection Reagent | QIAGEN | Cat#301705 |
| Human TGF-beta2 | PeproTech | Cat#100-35-2UG |
| Human TGF-beta3 | PeproTech | Cat#100-35-2UG |
| Cell-Light EdU Apoll 567 In Vitro Kit | RIBOBIO | Cat#C10310-1 |
| Glucose(GO)Assay Kit | SIGMA | Cat#SLCB1557 |
| Lactic Acid assay kit | Nanjing jiancheng | Cat#A019-2-1 |

**Table S3** The qPCR primers was showed as below:

| Gene | primer |
| --- | --- |
| PD-L1 | Forward Primer: TGGCATTTGCTGAACGCATTT  Reverse Primer: TGCAGCCAGGTCTAATTGTTTT |
| Thrombopoietin | Forward Primer: CTGAAGTGTTTCTCCCGAACAT  Reverse Primer: GCGGGTAGGCATACAGCAG |
| CXCL5 | Forward Primer: AGCTGCGTTGCGTTTGTTTAC  Reverse Primer: TGGCGAACACTTGCAGATTAC |
| EGF | Forward Primer: TGGATGTGCTTGATAAGCGG  Reverse Primer: ACCATGTCCTTTCCAGTGTGT |
| IGF | Forward Primer: GCTCTTCAGTTCGTGTGTGGA  Reverse Primer: GCCTCCTTAGATCACAGCTCC |
| TGF-β1 | Forward Primer: GGCCAGATCCTGTCCAAGC  Reverse Primer: GTGGGTTTCCACCATTAGCAC |
| PKM2 | Forward Primer: ATGTCGAAGCCCCATAGTGAA  Reverse Primer: TGGGTGGTGAATCAATGTCCA |
| LDHA | Forward Primer: ATGGCAACTCTAAAGGATCAGC  Reverse Primer: CCAACCCCAACAACTGTAATCT |
| GLUT1 | Forward Primer: GGCCAAGAGTGTGCTAAAGAA  Reverse Primer: ACAGCGTTGATGCCAGACAG |
| HK1 | Forward Primer: GCTCTCCGATGAAACTCTCATAG  Reverse Primer: GGACCTTACGAATGTTGGCAA |
| HK2 | Forward Primer: GAGCCACCACTCACCCTACT  Reverse Primer: CCAGGCATTCGGCAATGTG |
| GPI | Forward Primer: CAAGGACCGCTTCAACCACTT  Reverse Primer: CCAGGATGGGTGTGTTTGACC |
| PFKL | Forward Primer: GCTGGGCGGCACTATCATT  Reverse Primer: TCAGGTGCGAGTAGGTCCG |
| ALDOA | Forward Primer: ATGCCCTACCAATATCCAGCA  Reverse Primer: GCTCCCAGTGGACTCATCTG |
| PGAM1 | Forward Primer: GTGCAGAAGAGAGCGATCCG  Reverse Primer: CGGTTAGACCCCCATAGTGC |
| ENO1 | Forward Primer: AAAGCTGGTGCCGTTGAGAA  Reverse Primer: GGTTGTGGTAAACCTCTGCTC |
| ENO2 | Forward Primer: AGCCTCTACGGGCATCTATGA  Reverse Primer: TTCTCAGTCCCATCCAACTCC |
| PKM1 | Forward Primer: CGAGCCTCAAGTCACTCCAC  Reverse Primer: GTGAGCAGACCTGCCAGAC |
| 18S | Forward Primer: CCAGTAAGTGCGGGTCATAAG  Reverse Primer: GGCCTCACTAAACCATCCAA |
| si-SMAD2 | Forward Primer: GUCCCAUGAAAAGACUUAATT  Reverse Primer: UUAAGUCUUUUCAUGGGACTT |
| si-TGFB1 | Forward Primer: CAAGCAGAGUACACACAGCAU  Reverse Primer: AUGCUGUGUGUACUCUGCUUG |
